# Vitamin D and immunomodulation in early rheumatoid arthritis: A randomized double-blind placebo-controlled study

**DOI:** 10.1371/journal.pone.0178463

**Published:** 2017-06-05

**Authors:** Ilaria Buondonno, Guido Rovera, Francesca Sassi, Micol Maria Rigoni, Claudia Lomater, Simone Parisi, Raffaele Pellerito, Giovanni Carlo Isaia, Patrizia D’Amelio

**Affiliations:** 1 Department of Medical Science, Gerontology and Bone Metabolic Disease Section, University of Torino, Torino, Italy; 2 Rheumatology Unit, Ospedale Mauriziano, Torino, Italy; 3 Rheumatology Department, AOU Città della Salute e della Scienza di Torino, Torino, Italy; VU University Medical Center, NETHERLANDS

## Abstract

The aim of this study was to evaluate differences in T helper cell sub-types and osteoclast (OCs) precursors in peripheral blood between patients affected by early rheumatoid arthritis (eRA) and healthy controls. The effect of administration of cholecalcipherol on clinical and laboratory parameters was subsequently evaluated, by a parallel, randomized double blind, placebo controlled trial. Thirty nine eRA patients and 31 age-matched controls were enrolled and compared for levels of 25OH vitamin D, T helper cell sub-types, OCs precursors including both classical and non-classical and pro-inflammatory cytokines at baseline. Eligible patients were female ≥18 years of age with a diagnosis of RA, as defined by the American College of Rheumatology 2010 criteria for <6 months prior to inclusion in the study. Patients with auto-immune or inflammatory diseases other than RA were excluded. Patients treated with glucocorticoids (GCs), disease modifying activity drugs and biologic agents within the past 6 months were also excluded. In the second phase of the study, eRA patients were randomly assigned to standard treatment with methotrexate (MTX) and GCs with (21) or without (18) cholecalcipherol (300,000 IU) and followed for 3 months; the randomization was done by computer generated tables to allocate treatments. Three patients didn’t come back to the follow up visit for personal reasons. None of the patients experienced adverse events. The main outcome measures were T cells phenotypes, OCs precursors and inflammatory cytokines. Secondary outcome measure were clinical parameters. In eRA, 25OH vitamin D levels were significantly lower. CD4+/IFNγ+,CD4+/IL4+, CD4+/IL17A+ and CD4+IL17A+IFNγ+, cells were increased in eRA as well as non-classical OCs precursors, whereas T regulatory cells were not altered. TNFα, TGFβ1, RANKL, IL-23 and IL-6 were increased in eRA. Non-classical OCs, IL-23 and IL-6 correlated with disease severity and activity. Standard treatment with MTX and GC ameliorated clinical symptoms and reduced IL-23, whereas it did not affect CD4+ cells sub-sets nor OCs precursors. After 3 months, the combined use of cholecalcipherol significantly ameliorated the effect of treatment on global health. In eRA, a significant imbalance in T CD4+ sub-types accompanied by increased levels of non-classical OCs precursors and pro-inflammatory cytokines was observed. A single dose of cholecalcipherol (300,000 IU) combined with standard treatment significantly ameliorates patients general health.

## Introduction

Recent studies have focused on the role of hypovitaminosis D in the pathogenesis of rheumatoid arthritis (RA) [1] and an inverse relationship between serum levels of 25-hydroxyvitamin D and disease activity or functional impairment has been observed [2,3].

Recently, a pilot exploratory study demonstrated that supplementation of vitamin D is effective in ameliorating clinical outcomes in RA patients affected by hypovitaminosis D [4].

Despite these recent efforts, there are no intervention studies that have specifically evaluated the effect of vitamin D treatment on T helper (CD4+, Th) cell phenotypes, cytokine production and osteoclast (OCs) precursor cells in RA patients.

It is well known that RA is an immune-mediated disease characterized by T cell activation and bone erosions with articular inflammation and progressive joint destruction that severely reduces patients quality of life; nevertheless a clear role for different Th cells has not yet been established [5].

An imbalance in Th cell activation has been suggested as the main pathogenic features of RA, nevertheless, studies on the role for different Th phenotypes such as T regulatory cells (Tregs), Th1, Th2 and Th17, have obtained contradictory results [5].

Different studies on Treg phenotype and function in RA patients have obtained conflicting results [6–11], these cells may have a role in the control of bone erosions in RA as, in experimental models, are able to inhibit OCs formation and activity [12–13].

Some recent studies also suggest a role for increased Th1 cells in the pathogenesis of RA, in particular these cells being frequently observed in the synovial fluid of RA patients [14].

Th1 produces IFNγ that have controversial effects on OCs formation and activity [15–16], and TNFα that induces OCs formation in the presence of adequate levels of RANKL [17], RANKL being the cytokine mainly responsible for OCs formation and activity.

Th17 have also been investigated in RA and these cells have been shown to play an important role in experimental models of arthritis [5,18,19].

In RA patients, Th17 are recruited within the synovium where they exert pro-inflammatory and pro-osteoclastogenic effects [20,21].

Another Th subtype has been described in RA patients; this is a transitional cell that expresses both IFNγ and IL-17, and correlates with disease activity [22].

Taken together, these data suggest that both an increase in levels of Th1 and Th17 cells may be responsible for increased OCs formation and bone erosions in RA, whereas a role for reduced Tregs still remains controversial.

OCs have a critical role in RA and are required for bone erosions to occur [23].

OCs precursors circulate in the peripheral blood and express the following cell surface antigenic markers: CD14+/CD11b+ and CD51/61+ (classical OCs precursors) and CD14 and high levels of CD16 (CD14+/CD16^bright^, non-classical OCs precursors). Classical OCs precursors have been found to be increased in conditions characterized by increased bone resorption such as post-menopausal osteoporosis, bone metastases and other conditions; whereas non-classical OCs precursors has been observed during inflammatory diseases characterized by increased bone resorption [24–26].

How chronic inflammation affects bone resorption and different OCs precursors in RA is still largely unclear.

Despite the fact that studies have shown an immunomodulatory effect of vitamin D in animal models of RA [27], this is the first double-blind placebo-controlled pilot study that specifically examines the effect of vitamin D administration on Th phenotype, and OCs precursor cells in patients affected by early RA (eRA), moreover we evaluated the effect of cholecalcipherol on clinical signs and symptoms of eRA.

## Materials and methods

### Trial design

This is a parallel, randomized, placebo-controlled, double blind, trial (registered on 2010-03-29 in the European Clinical Trials Database (EudraCT) as OM-2009-01 EudraCT 2009-015835-34, available at https://www.clinicaltrialsregister.eu/ctr-search/trial/2009-015835-34/IT). The authors confirm that all ongoing and related trials for this drug/intervention are registered.

The randomization was done by computer generated tables to allocate treatments. The study was approved by the Ethical Committee of the A.O.U. Città della Salute e della Scienza—A.O. Ordine Mauriziano—A.S.L. TO1, Turin Italy (protocol number: OM-2009-001, on 2010-01-12) and informed consent was obtained from all participants.

Patients and controls were enrolled in the Rheumatology Unit, Ospedale Mauriziano, and in the Rheumatology Department, AOU Città della Salute e della Scienza di Torino, Torino, Italy. Lab experiments were performed in the bone biology lab of the Department of Medical Science, Gerontology and Bone Metabolic Disease Section, University of Torino, Torino, Italy, researcher were in blind as respect to diagnosis (healthy control or eRA) and to treatment allocation.

The study was divided into two phases: in the first phase, we compared T CD4+ phenotypes, OCs precursor cells and cytokines in the peripheral blood of women affected by eRA compared to healthy age-matched women.

The second phase of the study was a parallel, double-blind placebo-controlled randomized trial on the effect of 300,000 IU of cholecalcipherol on clinical features and experimental parameters of eRA patients.

### First phase: Baseline evaluation of patients and control subjects

Thirty nine women affected by eRA not previously treated with glucocorticoid (GC) or disease modifying activity drugs (DMARDs) and 31 healthy age-matched women were enrolled in the present study between 2010-07-05 and 2014-25-11.

Eligible patients were female ≥18 years of age with a diagnosis of RA, as defined by the American College of Rheumatology (ACR; formerly, the American Rheumatism Association) 2010 [28] criteria for <6 months prior to inclusion in the study.

Patients with a history of auto-immune or inflammatory diseases other than RA, tuberculosis, malignancy, renal, hepatic, hematologic, gastrointestinal, endocrine, pulmonary, cardiac, neurologic, or cerebral disease were excluded. Patients treated with GCs, DMARDs, biologic agents within the past 6 months were also excluded. Renal insufficiency was ruled out on the basis of clinical history and previous creatinine measure.

### Clinical evaluation of eRA

Disease activity and progression was evaluated using the Disease Activity Score 28 (DAS 28) [17]. This score assesses the number of swollen and tender joints and includes measurement of erythrocyte sedimentation rate (ESR).

Joint pain was evaluated by the Visual Analog Scale for Pain (VAS Pain) and the Health Assessment Questionnaire (HAQ) was used to evaluate the functional status (disability) [29]. Global Health (GH) was evaluated on a numeric scale (0–100, 0 worst, 100 best status). Basal levels of 25-OH vitamin D, ESR and C reactive protein (CRP) were measured in all subjects. ESR and CRP were not measured in healthy controls as their increase in not sensitive nor specific in healthy subject.

### Enumeration of T helper and osteoclast precursor cells by flow cytometry

Peripheral blood mononuclear cells (PBMCs) were obtained from all the surveyed groups with the Ficoll-Paque method from 40 ml peripheral blood in lithium heparin as previously described [17].

Flow cytometry was used to quantify osteoclast precursors and Th cell subsets in peripheral blood. We measured classical OC precursors identified by staining PBMCs (1×10^6^) with fluorescein isothiocyanate-conjugated (FITC)-conjugated anti-VNR, phycoerythrin-conjugated (PE)-conjugated anti-CD14 and allophycocyanin-conjugated (APC)-conjugated anti-CD11b mAb, or with the corresponding isotype control followed by incubation at 4°C for 30 min. Triple-positive (CD14+/CD11b+/VNR+) cells were considered as osteoclast precursors according to previous literature [17].

We also measured non-classical OC precursors stained with anti-CD14 (PE) and CD16 peridinin-chlorophyll protein-conjugated (PerCP) antibodies or with the corresponding isotype control followed by incubation at 4°C for 30 min. Cells positive for CD14 and expressing high levels of CD16 (CD16^bright^) were considered non-classical osteoclast precursors according to previous literature [25].

T helper cells were identified by T helper 1/2/17 phenotyping kit (BD Biosciences, Franklin Lakes, NJ, USA), according to manufacturer’s instructions. PBMCs (1×10^6^) were incubated in 6-well plates, with the cell cytokine secretion inhibitor, ionomycin (50ng/ml) and phorbol 12-myristate 13-acetate (1μg/ml) (as aspecific stimulus). After 5 hours, cells were collected and fixed with 1 ml Cytofix Buffer for 20 min at room temperature, then washed and re-incubated with 1 ml BD Perm/Wash Buffer for 20 min at room temperature.

T-helper lymphocytes were identified by incubating with monoclonal labelled antibodies cocktail (and corresponding isotype controls): anti-IL4 (APC-conjugated), anti-IL17 (PE-conjugated), anti-CD4 (PerCP) and anti-INF_γ_ (FITC-conjugated). Cells were washed and collected in 300 μl 1% PAF.

T regulatory (Treg) cells were identified by the “Human Regulatory T-cell Staining Kit” (eBioscience Inc. San Diego, CA, USA) in accordance with manufacturer’s instructions. Briefly, the following labelled monoclonal antibodies and corresponding isotype controls were used: anti-CD4 (FITC-conjugated); anti-CD25 (APC-conjugated); and anti-FOXP3 (PE-conjugated). After surface staining for CD4 and CD25 for 20 min at room temperature, cells were washed and fixed with the fixative and permeability solution (Fix-Perm Buffer) and incubated with rat serum to remove aspecific binding sites. Cells were then incubated with anti-FoxP3 (PE-conjugated), as intra-nuclear staining, and collected in 300 μl 1% PAF. Flow cytometry was performed by FACS Calibur flow cytometer and Cell Quest Software (BD Biosciences) and each analysis consisted of at least 100,000 events recorded within the lymphocyte gate.

### Measurement of cytokine production

In order to evaluate the activity of various T cell subtypes and to study cytokines involved in OCs formation we measured the levels of TNFα, IFNγ, TGFβ1, IL-4, IL-17, IL-23, IL-6, OPG (R&D Duoset, Minneapolis, MN, USA), and total RANKL (BioVendor R&D, Brno, Czech Republic) by the ELISA technique in serum.

### Second phase: Therapeutic intervention

After baseline evaluation, eRA patients were randomly allocated to treatment with: methotrexate (MTX) 15 mg/week (im or sc) and methylprednisolone (GC) per os 2–4 mg/day plus cholecalciferol 300,000 IU (N = 21) or placebo (N = 18) in single administration at the baseline evaluation (cholecalcipherol was kindly provided by Abiogen Pharma S.p.A, Pisa Italy), healthy controls do not participate in this phase of the study. Levels of 25-OH vitamin D were measured at the end of the study, the use of 300000 UI of cholecalcipherol was decided regardless to basal level of 25-OH vitamin D and starting from the hypothesis of the presence of vitamin D insufficiency in eRA patients, according to previous literature [1–3]. The use of 300000 UI was choose according with Premaor MO and coll. [30], they showed that, in the short term, a single 300 000 IU oral dose of vitamin D was more effective than 800 IU per day to increase serum 25(OH)D levels. Randomization was done by the principal investigator, patients were enrolled by participants in the study, lab measurement and statistical analyses were done in blind to treatment.

**Fig 1:**
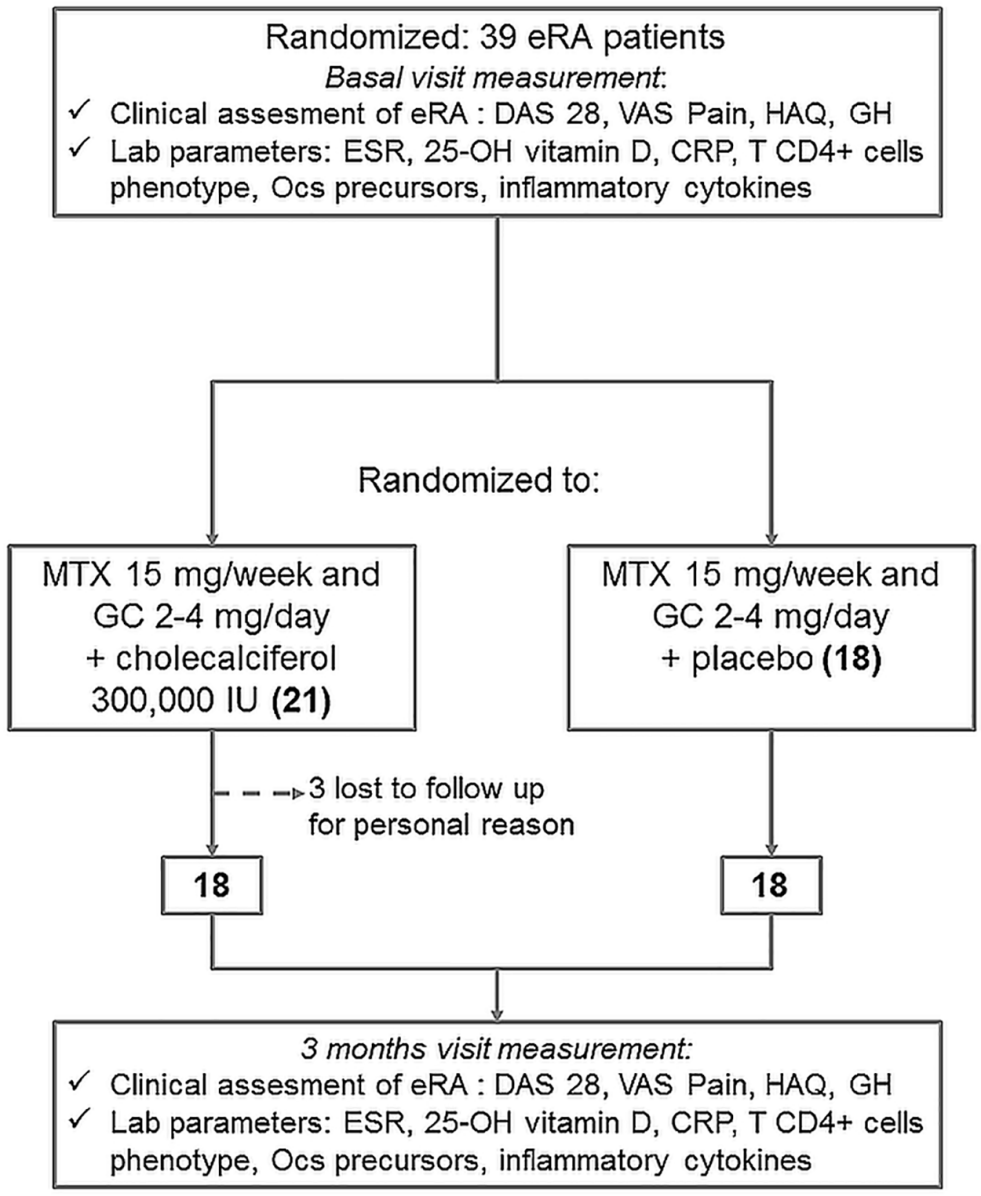
The diagram shows the study design and the number of patients at each visit in bold, the tests performed at each visits are specified.

The use of nonsteroidal anti-inflammatory drugs (NSAID) and paracetamol for pain relief was allowed and recorded at the control visit as “less than 7 day a week” or “daily”. After three months of therapy, patients were recalled to the centre and baseline evaluations were repeated, 3 patients did not come back to the follow up visit due to personal problems (Fig 1), physician were asked to register any adverse event, and to judge if such event may be related to cholecalcipherol. None of the patients experienced adverse events. Data from patients dropped out were considered for the comparison with healthy controls, but not on the second part of the study.

The primary outcome measures of the first phase of the study were differences in Th subtypes and OCs precursors between patients and healthy controls.

The primary outcome measures of the second phase of the study were changes in number of Tregs, secondary outcome measures were changes in number of other CD4+ cells, OCs precursors and clinical parameters.

### Statistical analysis

Patients at baseline and controls were compared for the analysed variables by one-way ANOVA for Gaussian variables and by Mann Whitney test for non-Gaussian variables. T cell sub-sets, OCs precursors and cytokines were correlated with clinical parameters by means of Spearman coefficient correlation. To evaluate a possible effect of cholecalcipherol on clinical parameters and on laboratory variables significantly affected by eRA a multivariate analysis of variance (MANOVA) for repeated measures was used after logarithmically transforming non-Gaussian variables. The sample size of the second phase of the study provided an 80% power, assuming a two-sided significance level of 0.05, to detect differences greater than 1.5% in Tregs (t-test). Tregs were chosen as key variables on the basis of previous intervention trial in healthy volunteers [28].

Statistical analysis was performed using SPSS 21.0 for windows. Graphs were drawn using Graph Pad 7.0 for windows and a p-value <0.05 was considered statistically significant.

## Results and discussion

### Clinical characteristics of patients and controls

Patients and control subjects were not significantly different for age, post-menopausal period and body mass index (BMI), whereas levels of 25-OH vitamin D were significantly lower in patients compared to controls (Table 1).

**Table 1:** Clinical characteristics of patients and controls. Mean ± SD is shown for Gaussian variables, for non-Gaussian variables (indicated by *) median (25–75 percentiles) are indicated. P values were calculated by means of ANOVA one-way for Gaussian variables and by means of Mann-Whitney test for non-Gaussian ones.

	eRA (39)	Controls (31)	p
**Age (yrs)**	54±8	55±13	0.743
**post-menopausal period (yrs)**	5±9	6±9	0.076
**BMI**	26.4±6.1	25.7±5.3	0.602
**25OH-vitamin D (ng/mL)**	16±2.1	26±2.23	**0.002**
PTH (pg/mL)*	38.15 (25.9–61.8)	32.9 (25.9–50.5)	0.545
**Creatinine (mg/dL)**	0.60±0.4	0.59±0.35	0.958
**DAS 28**	5.7±0.9	ND	
**HAQ**	1.3±0.8	ND	
**NRS**	62.9±20.2	ND	
ESR*	38 (22.3–57.8)	ND	
CRP (mg/L)*	10.0 (4.0–25.0)	ND	
**GH**	50.1±17.1	ND	

### T helper cell subsets in eRA

**Fig 2:**
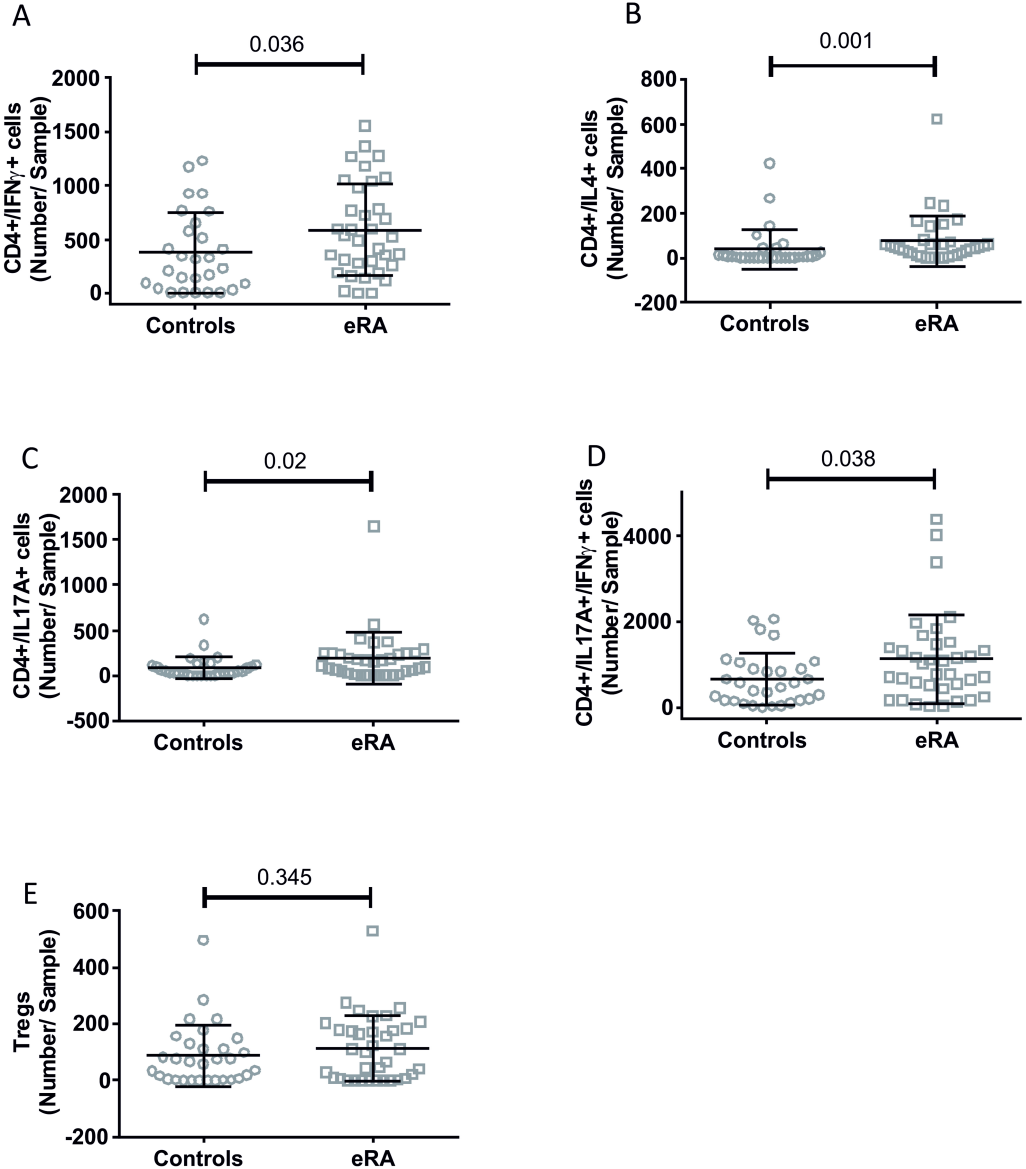
T helper subsets in eRA patients and healthy controls. Dot plots show CD4+/IFNγ+ (panel A), CD4+/IL4+ (panel B), CD4+/IL17A+ (panel C), CD4+/IL17A+/IFNγ+ (panel D) and Tregs (panel E);mean and SD are shown, p values were calculated by Mann-Whitney test and are displayed.

In patients affected by eRA, T helper subsets were significantly altered. In particular, we found an increase in in the number of CD4+/IL17A+, CD4+/IFNγ+, CD4+/IL4+ and CD4+/IL17A+/IFNγ+ lymphocytes, whereas Tregs were not significantly affected (Fig 2). CD4+/IL17A+,cells were the only T h cells correlated with a clinical parameter: CRP (r = 0.35, p = 0.04).

### OCs precursor cells in eRA

**Fig 3:**
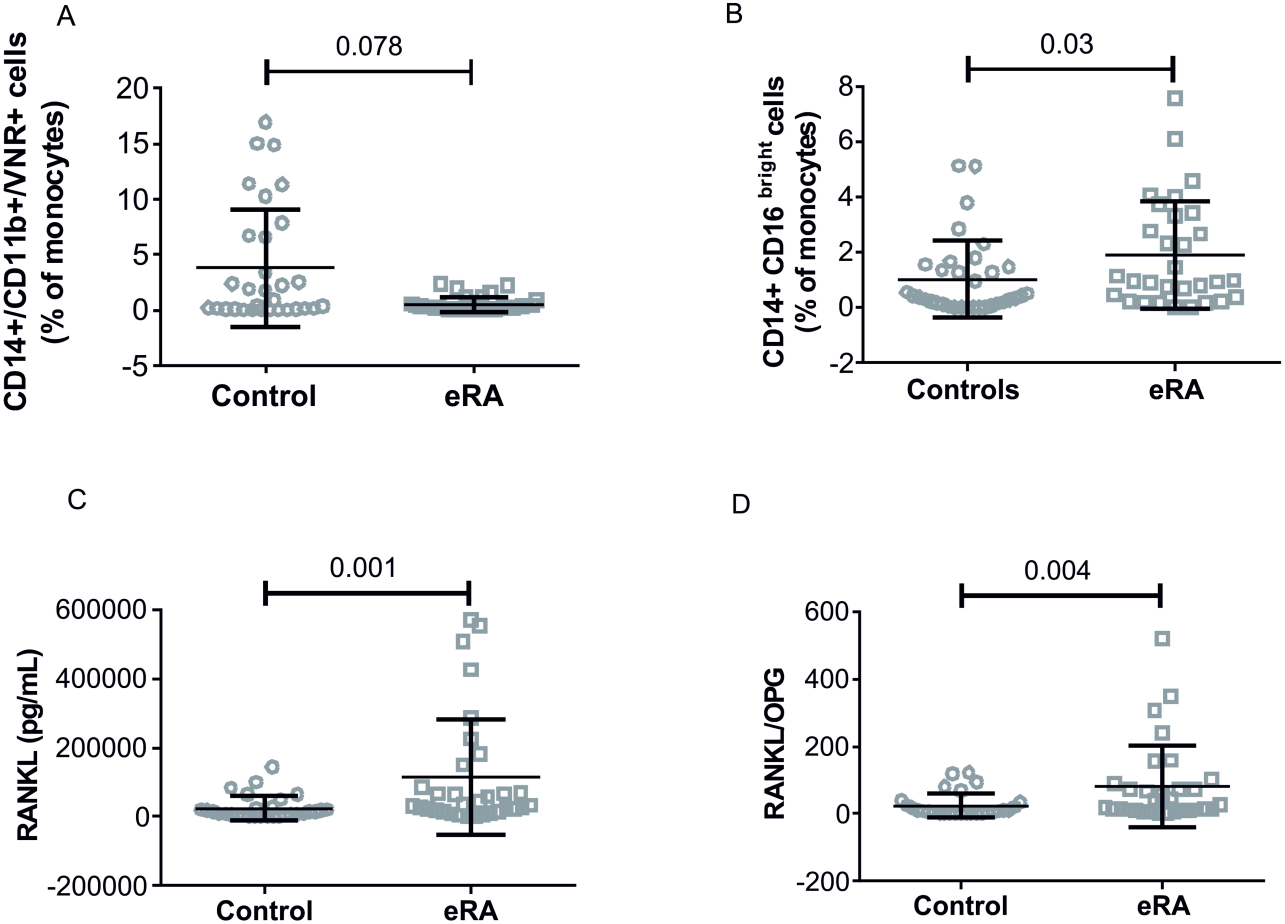
OCs precursors in eRA patients and healthy controls. Dot plots show classical OCs precursor (panel A), non-classical OCs precursor (panel B), RANKL (panel C) and RANKL/OPG (panel D); mean and SD are shown, p values were calculated by Mann-Whitney test and are displayed.

In order to evaluate the role of OCs precursor cells in bone damage, we measured both classical and non-classical OCs precursors in PBMCs from eRA and controls. Classical OCs were not increased in eRA patients whereas non-classical OCs were significantly increased (Fig 3A and 3B). Interestingly, non-classical OCs precursors, but not classical were directly correlated with DAS28 score (r = 0.36, p = 0.033) and with CRP (r = 0.37, p = 0.033).

### Inflammatory cytokines in eRA

Levels of RANKL were significantly increased in eRA vs controls while OPG was not affected and, as a consequence, the RANKL/OPG ratio was significantly increased (Fig 3C and 3D). Furthermore, RANKL/OPG ratio was directly correlated with levels of non-classical OCs precursor cells (r = 0.36, p = 0.04).

**Fig 4:**
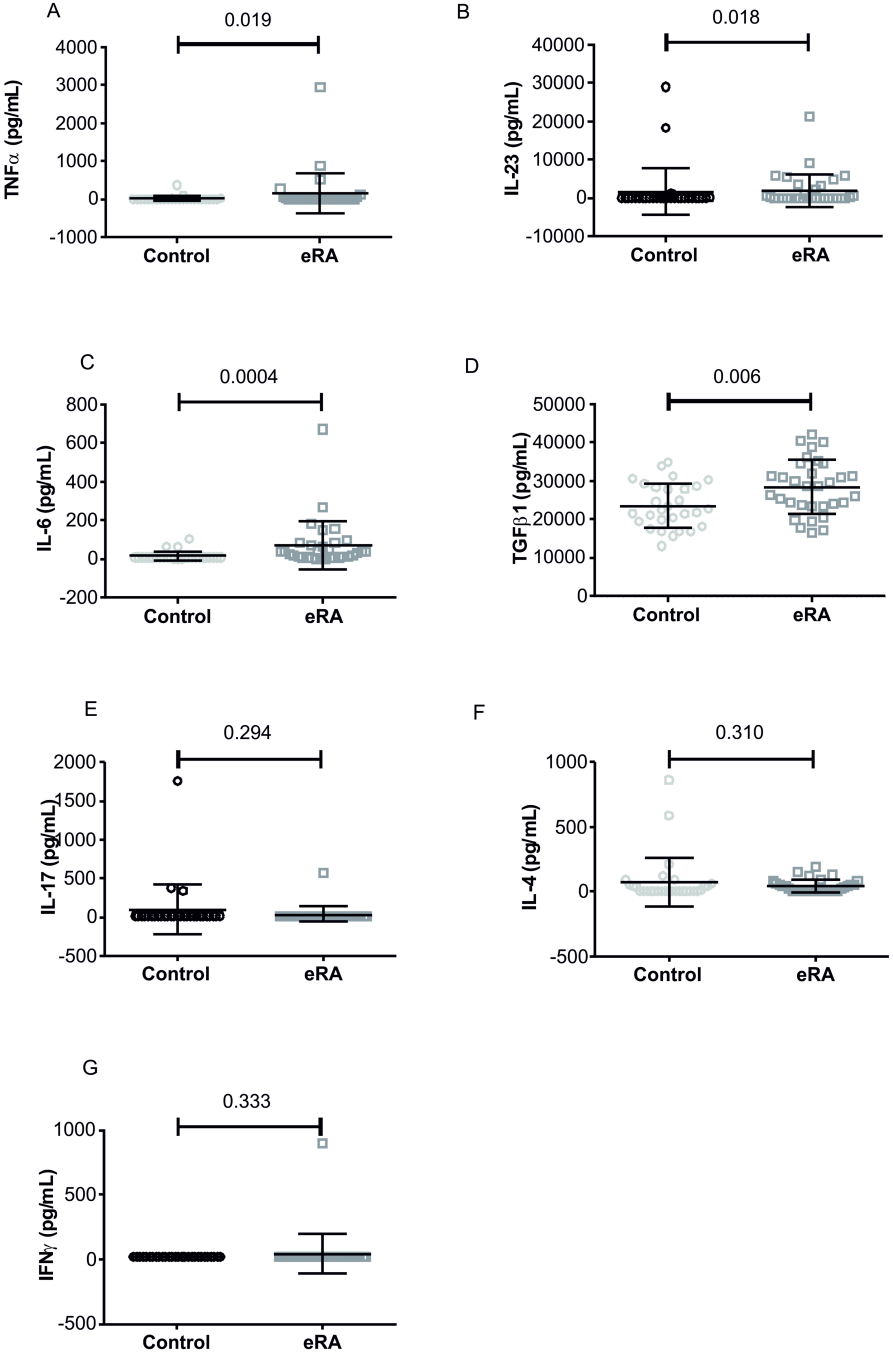
Inflammatory cytokines in eRA patients and healthy controls. Dot plots show classical IL-23 (panel A), IL-17 (panel B), IL-4 (panel C), IL-6(panel D), TGFβ1 (panel E), TNFα (panel F) and IFNγ (panel G); mean and SD are shown, p values were calculated by Mann-Whitney test and are displayed.

Analysis of other inflammatory cytokines in the peripheral blood of patients revealed a significant increase in TNFα, TGFβ, IL-23 and IL-6, whereas IL-17 and IFNγ levels were not significantly different in patients compared to controls (Fig 4). IL-23 and IL 6 were significantly correlated with clinical parameters. In particular, IL-23 was positively correlated with ESR (r = 0.36, p = 0.04), IL-6 with CRP (r = 0.39, p = 0.03) and DAS28 (r = 0.4, p = 0.02).

### Effect of vitamin D on GH in eRA patients

To evaluate the possible effect of vitamin D treatment on clinical signs and symptoms of eRA, on T cells differentiation and function, on inflammation and osteoclastogenesis, we randomly assigned eRA patients to receive 300,000 IU of cholecalcipherol or placebo, in addition to standard treatment. Three months after treatment levels of 25-OH vitamin D were significantly increased in patients treated with cholecalcipherol (p = 0.015), whereas remained stable in patients treated with placebo (p = 0.08, Table 2) as expected. Patients receiving standard treatment plus colecalcipherol or placebo had similar clinical characteristics (Table 2).

**Table 2:** Clinical characteristics of patients treated with standard treatment with or without cholecalcipherol 300000 IU. Mean ± SD is shown for Gaussian variables, for non-Gaussian variables (indicated by *) median (25–75 percentiles) are indicated. Comparison between the two groups after intervention were calculated by means of ANOVA one-way after Bonferroni correction, p values are shown where appropriate.

	Placebo (18)	Cholecalcipherol 300000 IU (18)	p
**Age (yrs)**	54±12	56±14	
**post-menopausal period (yrs)**	6±9	5±9	
**BMI**	25.5±5.6	26.0±5.0	
**Basal 25OH-vitamin D (ng/mL)**	16±2.1	16±4	
**Three month 25-OH vitamin D (ng/mL)**	18±3.4	28.7±4.3	**0.01**
**Basal PTH (pg/mL)**	37.1 (24.2–71.15)	43.5 (32.9–52)	
**Three months PTH (pg/mL)**	47.2 (41.5–60.0)	32.6 (21.1–50.5)	0.200
**Basal creatinine (mg/dL)**	0.35±0.24	0.7±0.4	
**Three months creatinine (mg/dL)**	0.3±0.2	0.45±0.3	0.288
**DAS 28**	5.8±0.9	5.6±0.9	
**HAQ**	1.4±0.7	1.3±1.0	
**VAS pain**	68.3±18.9	57.5±20.6	
ESR*	38 (23.5–59)	38 (17.5–60.0)	
CRP (mg/L)*	12.35 (5.5–23.5)	10.0 (2.5–31.0)	
**GH**	50.0±16.5	50.3±18.2	

**Fig 5:**
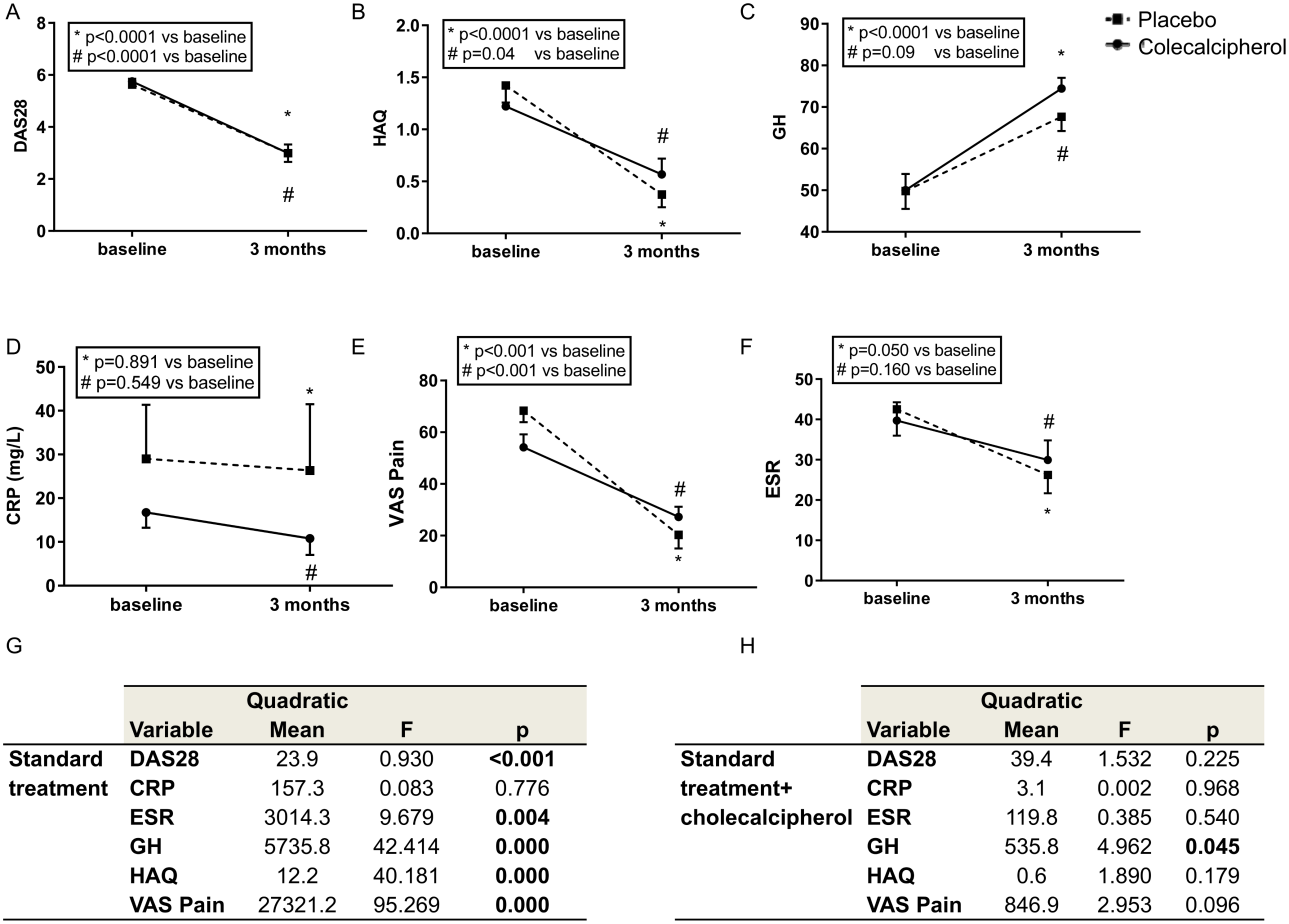
Effect of treatment with and without cholecalcipherol on clinical parameters in eRA patients. Graphs show DAS28 (panel A), HAQ (panel B), GH (panel C), CRP (panel D), VAS pain (panel E) and ERS (panel F); mean and SD are shown, p values were calculated by Student paired t-test and are displayed. Tables (panels G and H) show the MANOVA for repeated measures for DAS28, HAQ, GH, CRP, VAS pain and ESR and the effect of standard treatment with or without the addiction of cholecalcipherol, quadratic mean, F and p value are shown. All the analyses were done in 18 patients per group. P values were corrected for multiple comparisons.

For clinical parameters, standard treatment significantly reduced DAS28, CRP, ESR and VAS pain and improved GH and HAQ. Furthermore, the association of vitamin D to standard treatment was more effective in ameliorating GH, whereas it had no effect on the other parameters (Fig 5). As regards the use of NSAID and paracetamol it was equally distributed in the two groups, in the placebo group 3 patients (17%) used NSAID or paracetamol less than 7day a week, whereas 15 (83%) used NSAID or paracetamol daily. In the group treated with cholecalcipherol 6 patients (33%) used NSAID or paracetamol less than 7day a week, whereas 12 (67%) used NSAID or paracetamol daily (χ2 = 2.62, p = 0.270).

Standard treatment with or without the addition of vitamin D had no significant effect on T helper subset and on OCs precursor cells (Table 3). T helper cells and OCs were not significantly different between eRA treated with standard treatment or with standard treatment plus cholecalcipherol (data not shown).

**Table 3:** The table shows the effect of standard treatment compared with standard treatment plus cholecalcipherol 300000 IU on CD4+/IFNγ+, CD4+/IL17A+, CD4+/IL4+, CD4+/IL17A+/ IFNγ+ and non-classical OCs precursors (these variables were significantly different between patients and controls). The variables were transformed in logarithm as they were distributed according to a non-Gaussian curve. Mean±SE, quadratic mean (Q), F and p values were calculated by MANOVA for repeated measures. P values were corrected for multiple comparisons. P values are shown for comparison between different treatment (*) and within treatment (baseline vs 3 months **).

	Variable	Mean±SE basal	Mean±SE 3 months	p**	Q	F	p*
**Treatment (18)**	CD4+/IFNγ	1.3±0.3	2.6±0.7	0.095	31.41	3.5	0.071
	CD4+/IL4+	0.3±0.1	0.2±0.06	0.917	0.021	0.166	0.687
	CD4+/IL17A+	0.6±0.3	1.2±0.5	0.391	1.933	1.112	0.301
	CD4+/IL17A+/ IFNγ+	0.8±0.3	2.2±0.5	0.238	8.041	0.256	0.617
	Non-classical OCs	0.1±0.04	0.1±0.04	0.168	39.84	2.071	0.161
Treatment* Colecalcipherol (18)	CD4+/IFNγ	2.0±0.4	2.3±0.7	0.403	3.600	0.403	0.531
	CD4+/IL4+	0.2±0.1	0.2±0.1	0.552	0.004	0.030	0.863
	CD4+/IL17A+	0.6±0.3	0.4±0.5	0.472	5.346	3.077	0.090
	CD4+/IL17A+/ IFNγ+	1.2±0.3	0.7±0.5	0.324	82.34	2.622	0.117
	Non-classical OCs	0.1±0.04	0.1±0.04	0.928	26.63	1.384	0.249

**Fig 6:**
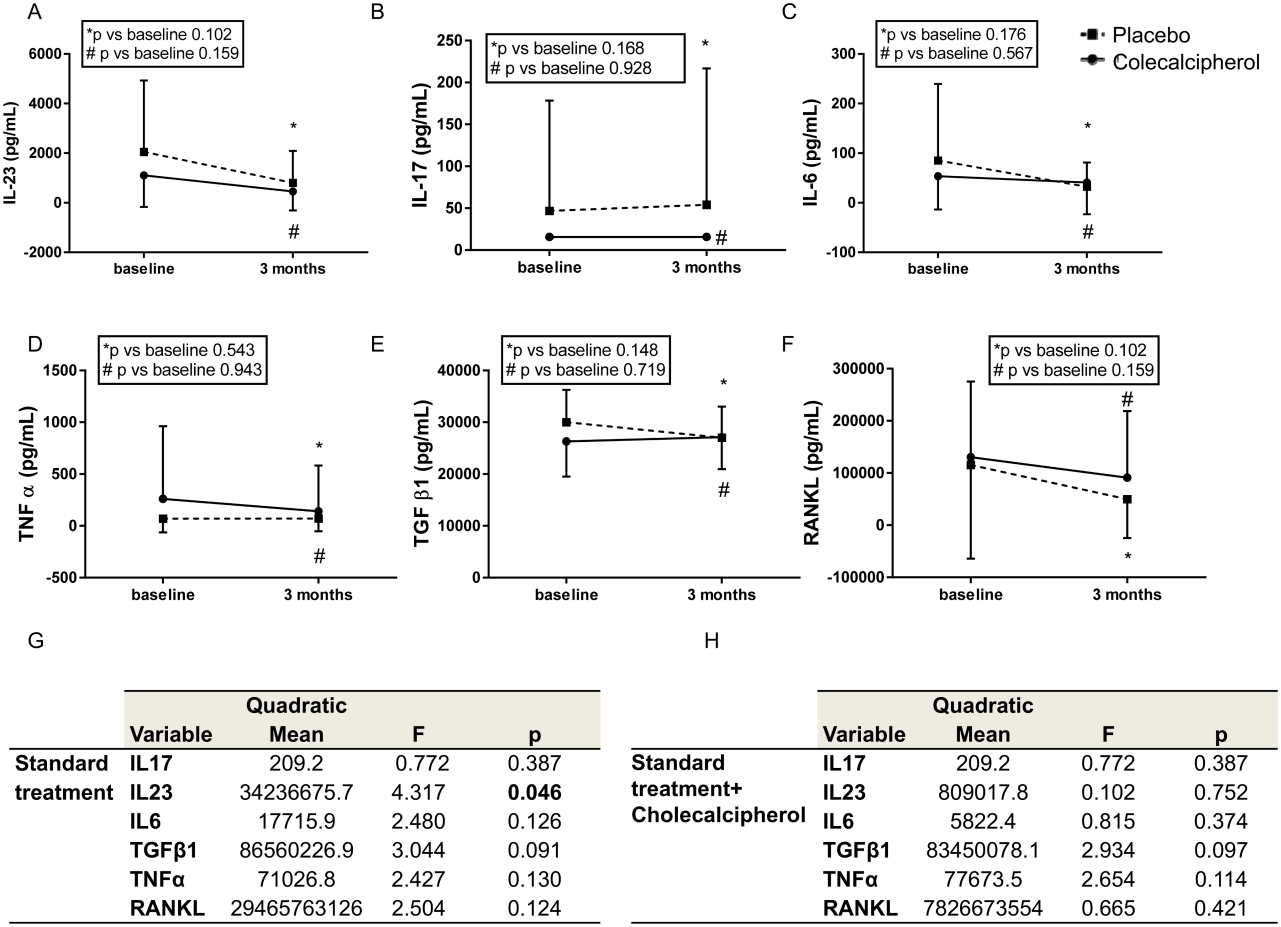
Effect of treatment with and without cholecalcipherol on inflammatory cytokines in eRA patients. Graphs show IL-23 (panel A), IL-17 (panel B), IL-6 (panel C), TNFα (panel D), TGFβ1 (panel E) and RANKL (panel F); mean and SD are shown, p values were calculated by Mann-Whitney test and are displayed. Tables (panels G and H) show the MANOVA for repeated measures for IL-23, IL-17, IL-6, TNFα, TGFβ1 and RANKL and the effect of standard treatment with or without the addition of cholecalcipherol, quadratic mean, F and p value are shown. The linear model was carried out after logarithmic transformation of non-Gaussian variables. All the analyses were done in 18 patients per group. P values were corrected for multiple comparisons.

Regarding inflammatory and pro-osteoclastogenic cytokines, standard treatment had a significant effect in reducing IL-23, whereas the addition of vitamin D had no significant effect (Fig 6).

Data on treatment were obtained in 18 eRA patients per group (standard treatment with or without cholecalcipherol).

In this paper, we evaluated RA patients at the very beginning of their disease, therefore permitting us to investigate early immunological alterations in RA. Our data revealed a significant increase in levels of CD4+/IFNγ+ and CD4+/IL17A cells in the peripheral blood of eRA patients compared to age- and gender-matched control subjects. This finding supports previous literature on more advanced RA showing increased homing of CD4+/IFNγ+ and CD4+/IL17A cells in the inflamed synovium [31,32] and suggests that, during the early phase, levels of these cells are increased in the peripheral blood and may migrate to the synovium only in the more advanced phase. Moreover, CD4+/IL17A cells detected in the peripheral blood are directly correlated to severity of inflammation, as measured by CRP levels, thus confirming their involvement in the pathogenesis of the disease.

We did not find a significant imbalance in Tregs, on this regard, previous studies have found conflicting results as some papers described a decrease in Tregs [6–8], whereas others did not observe this decrease [9,10] or found an increase [11]. This discrepancy largely depends on differences in the study design [5]. In our study, we found no significant difference in levels of Tregs in the peripheral blood of eRA patients suggesting that the *primum movens* of RA is not due to a decreased ability of the immune system to control inflammation, but to an increase in T cell activation *per se*. This observation is also reinforced by the increased levels of TGFβ1, suggesting an increased reaction of Tregs against auto immunity in these patients. In addition, CD4+/IL4+ cells were modestly increased in our patients thus suggesting an early imbalance also in these Th cells; other studies suggested a decrease of Th2 in more advanced forms of RA, our data suggest that this decrease may be more a consequence of the treatment rather than a cause of the disease. According to this hypothesis a recent paper on untreated eRA showed an increase in Th2 cells in peripheral blood [11].

A transitional cell type CD4+/IL17A-IFNγ has been previously described in RA [21] in particular at the level of the synovium. We found a significant increase in the number of these cells in the peripheral blood of eRA patients, that may later migrate within the inflamed synovium.

We also observed a significant increase in levels of non-classical OCs in eRA, whereas classical ones were not significantly different in patients and controls. Non-classical OCs precursors correlates with the grade of inflammation and the severity of the disease, suggesting that these cells, more than classical OCs, may be responsible for articular erosions in eRA patients. Moreover RANKL is increased in eRA and significantly correlates with these cells confirming a direct involvement of these precursors in RA bone damage.

With regard to inflammatory cytokines, our results confirmed previous studies showing increased levels of IL-6, TNFα and RANKL [33] also in the early phase of RA. Interestingly, we also observed increased levels of IL-23 and TGFβ1.

IL-23 is required for the amplification and stabilization of CD4+/IL17A+ cells and an increase in levels of IL-23 may explain the increased formation of CD4+/IL17A+ [31]. Previous studies have demonstrated that levels of IL-23 are elevated in the serum and synovial fluid of patients with established RA, and these levels positively correlated with levels of disease severity [34,35]. In our study, we observed an increase in IL-23, recognised as occurring earlier than that the described increase in IL-17 [36].

TGFβ1 modulates anti-inflammatory and immunosuppressive responses and plays a key role in self-tolerance [37]. TGFβ1 also plays a complex role in the regulation of bone turnover and has been shown to cause both bone loss and gain in mice [38].

A recent study conducted in RA patients showed a non-significant increase in this molecule in the serum of patients compared to healthy controls [39], however our study is the first to evaluate levels of TGFβ1 in the early RA phase. We showed a significant increase in TGFβ1 that may have a compensatory effect in response to increased inflammatory state.

Regarding the possible role of vitamin D status in the development of RA, we confirmed findings from previous studies on the association between RA and lower levels of 25-OH vitamin D. The administration of standard treatment plus 300,000 IU of cholecalcipherol in this population was able to ameliorate their global health, but had no significant effect on specific signs and symptoms of RA, CRP, VAS pain and DAS 28, that were decreased following standard treatment. A previous open-label study in patients affected by RA showed that the administration of cholecalcipherol over a period of 3 months in patients with active disease ad hypovitaminosis contributed to a significant improvement in disease activity [4]. In our study we did not observe any significant effect of cholecalcipherol on DAS28. This may be due to the different study setting since we used different doses of cholecalcipherol: we administered 300,000 IU of cholecalcipherol as a single dose and evaluated the effect after three month. In contrast, the study by Chandrashekara and Patted, patients with active RA and hypovitaminosis were enrolled and treated with 60.000 IU/week for 6 weeks, followed by 60,000 IU/month for a total duration of 3 months [4].

Vitamin D receptor (VDR) gene is widely expressed in a variety of human cell types suggesting that vitamin D has, via the VDR, a far wider physiological function than the control of calcium homeostasis and bone remodeling. As regards immune system cells of the innate and adaptive immune system, such as monocytes, T and B cells and monocytes express VDR and are responsive to vitamin D (see for a review [40]). Here we focused on T cells due to their important role in the pathogenesis of RA [5] and considering experimental evidence that points towards an effect of vitamin D in regulating T cell proliferation, differentiation in different subsets and cytokine production (see for a review [40]). In our study, we found no significant effect of cholecalcipherol administration on T helper subsets, nor on cytokines production. Previous studies have shown that 1,25OH cholecalcipherol inhibits Th1 cytokines production [41], and promotes Th2 cytokines [42]. More recently it has been shown that 1,25OH cholecalcipherol reduces the differentiation of Th towards CD4+/IL17A+ markers and their secretion of pro-inflammatory cytokines (IL-17A, IFNγ) and induces differentiation towards Tregs [43].

Nonetheless, we found no significant effect of cholecalcipherol on T helper cells in eRA in our study. A recent study in humans affected by chronic autoimmune thyroiditis showed that oral cholecalcipherol is able to reduce IFNγ+ and IL-17+ Th cells [44] and to increase the expression Tregs [45]; in patients with type 1 diabetes mellitus the administration of oral cholecalcipherol was able to increase the suppressive capacity of Tregs within 12 months of treatment [46]. The majority of previous studies on the effect of vitamin D on T cells has been performed in vitro and using 1,25-OH cholecalcipherol. Human studies have been undertaken in different disease states using different doses of vitamin D, therefore, it is very difficult to compare the results. Corroborating our findings, a recent double-blind placebo-controlled study performed in hemodialysis patients showed no effect of oral cholecalcipherol on Th cell subsets [47]. The present report is the first human study evaluating the effect of cholecalcipherol administration in eRA patients. Also standard treatment with MTX has no effect of Th cells subsets, previous studies showed an effect of MTX on activated T cells in vitro [48, 49], whereas in a recent paper patients treated with MTX administered orally in a dose ranging from 7 to 25 mg has no decrease in peripheral T cells count [50]. Our data confirm no effect of MTX in combination with GCs of different T cells sub-types. As regards the effect of MTX on OCs it has been recently demonstrated that it inhibits osteoclastogenesis by reducing RANKL-induced calcium influx into OC precursors [51], however these data were obtained in vitro, whereas no data on humans in vivo are available. Here we show no effect of cholecalcipherol on classical and non-classical OCs precursors, although it has previously been shown that 1,25OH cholecalcipherol is effective in reducing CD16 expression in monocytes from asthmatic patients in vitro [52], whereas in hemodialysis patients, no effect on CD16+ cells was observed following treatment with oral cholecalcipherol [47].

We enrolled only women in this study as RA is more frequent in this gender, however this could be a limitation of the study, also the inclusion of patients regardless their basal 25-OH vitamin D levels could be a limitation of the study as the effect of cholecalcipherol may be greater in patients affected by more severe forms of hypovitaminosis D. Nevertheless the result of our study may be generalized to women with eRA especially considering the absence of adverse events, probably the administration of cholecacipherol may be more effective in patients with lower levels of 25OH vitamin D as also shown by Chandrashekara and Patted [4].

## Conclusion

In conclusion, we show that eRA patients significantly differ from age and sex matched controls for 25-OH vitamin D levels, Th cell subsets with increased CD4+/IFNγ+, CD4+/IL4+, CD4+/IL17A+ and CD4+/IL17A+/ IFNγ+ cells, and increased non classical OCs precursors. We also observed an increase in TNFα, TGFβ1, RANKL, IL23 and IL-6. Among experimental parameters non-classical OCs, IL-23 and IL-6 correlated with disease severity and disease activity.

Since it is known that standard treatment with MTX and GC is able to ameliorate clinical symptoms, here we show that standard treatment is effective in reducing IL-23, whereas it does not affect Th subset nor OCs precursors. However, the combined use of 300,000 IU of cholecalcipherol significantly ameliorates the effect of treatment on global health in eRA patients.

## Supporting information

S1 File Consort check list 2010. Consort check list for the study.
(PDF)

S2 File Study protocol approved by Ethical committee (Italian). Protocol of the study approved by the Ethical committee in original language (Italian).
(PDF)

S3 File Study protocol approved by Ethical committee (English). Protocol of the study approved by the Ethical committee in English language.
(PDF)
