# Supplementary material for: Vitamin D and immunomodulation in early rheumatoid arthritis: A randomized double-blind placebo-controlled study
Source: PLoS One. 2017 Jun 5;12(6):e0178463. doi: 10.1371/journal.pone.0178463 (PMC5459341; doi:10.1371/journal.pone.0178463)
Supplement: S2 File — Protocol of the study approved by the Ethical committee in original language (Italian). (PDF) [file pone.0178463.s002.pdf]

EFFETTO DELLA VITAMINA D SUI LINFOCITI T E SULL'OSTEOCLASTOGENESI NELL'ARTRITE REUMATOIDE.

Abstract

L'artrite reumatoide (AR) è una malattia immunomediata, caratterizzata da un'infiammazione articolare da cui deriva un danno tissutale. Nell'AR si verifica un'iperplasia del tessuto sinoviale che evolve verso una progressiva distruzione delle articolazioni. Studi radiologici dimostrano che nell'AR l'erosione ossea si verifica già negli stadi precoci di malattia e progredisce rapidamente. L'erosione conduce a severa deformità delle articolazioni colpite ed influisce negativamente sulla qualità di vita dei pazienti. Le alterazioni del sistema immunitario nell'AR sono complesse e non ancora completamente conosciute: è noto che i linfociti T maggiormente coinvolti sono i T helper (Th)1, i Th17 sono aumentati ed i linfociti T regolatori (T reg) sono diminuiti. Inoltre si osserva un aumento dell'attività degli osteoclasti sia a livello delle articolazioni infiammate, sia a livello sistemico. Recenti studi indicano che la 1,25 diidrossivitamina D3 (1,25(OH)2D3), la forma attivata della vitamina D, esercita effetti sulla crescita e sul differenziamento di molti tipi cellulari, ed ha capacità immunomodulanti. E' stato ipotizzato che la 1,25(OH)2D3 possa regolare direttamente la funzione dei linfociti T e B, ed influenzare il fenotipo e la funzione delle cellule dendritiche, promuovendo meccanismi di tolleranza ed una maggiore espressione di linfociti T regolatori.

Il ruolo della vitamina D nella patogenesi e nel trattamento dell'AR è ancora controverso. Alcuni studi evidenziano una correlazione tra scarso introito di vitamina D e AR, mentre altri ne ipotizzano un possibile utilizzo terapeutico.

Gli obiettivi dello studio sono: 1-definire il ruolo della vitamina D nell'eziopatogenesi dell'AR- 2-valutare come la vitamina D possa agire da agente immunomodulante nell'AR in fase precoce. 3-valutare gli effetti della vitamina D sull'osteoclastogenesi nelle fasi precoci di malattia 4-valutare un potenziale utilizzo terapeutico della vitamina D nella fasi precoci dell'AR

Esperimenti in vitro

Per verificare se i linfociti T di pazienti con AR siano ugualmente responsivi alla vitamina D rispetto ai controlli, paragonabili per sesso ed età, misureremo il VDR con la PCR ed il western bolt nei PBMC frazionati (linfociti T, linfociti B e monociti) e non, sia nei pazienti con AR che nel gruppo di controllo. Per determinare se i PBMC di pazienti con AR, con rispetto a quelli di donatori sani, siano in grado di produrre e/o inattivare la vitamina D nello stesso modo, valuteremo la capacità dei PBMC frazionati e non d'idrossilare la vitamina D 25OH in posizione 1 per ottenere il

metabolita attivo 1,25OH e la capacità di queste cellule di inattivare la vitamina D mediante un'idrossilazione in posizione 24. I metaboliti saranno misurati con HPLC dopo un breve periodo d'incubazione con la vitamina D 25OH e l'1,25OH.

Esperimenti in vivo

Arruoleremo nello studio in vivo 50 donne affette da AR in fase precoce di malattia, non precedentemente trattate con glucocorticoidi o farmaci modulanti l'attività di malattia e 50 controlli paragonabili per età.

In tutti i soggetti sarà misurato il livello basale di vitamina D 25 OH nel siero e sarà somministrato un questionario sull'introito di vitamina D con la dieta, tramite software PROGEO Photo Intake.

Per quantificare i precursori degli osteoclasti e le sottopolazioni di linfociti T nelle PBMC utilizzeremo la citofluorimetria. Per definire le caratteristiche fenotipiche dei linfociti T h misureremo il diverso pattern di produzione citochinica ed i markers dei Th1/Th2/Treg/Th17 con la real time PCR. Per valutare la produzione di citochine da parte dei PBMC e per individuare i tipi cellulari coinvolti, utilizzeremo tecniche ELISA e ELISPOT. Ricercheremo una possibile correlazione tra linfociti T, livelli basali di 25OH vitamina D ed osteoclastogenesi. Per valutare il ruolo dei linfociti T nel metabolismo osseo andremo anche a misurare i livelli di RANKL ed OPG prodotti in coltura. Queste citochine saranno messe in relazione con l'attività osteoclastogenica dei PBMC, con le diverse sottopopolazioni di linfociti T e con i livelli di 25OH vitamina D. Inoltre valuteremo la differenza dei parametri analizzati tra pazienti e controlli

Nella seconda parte dello studio, le pazienti saranno, randomizzate ad uno specifico trattamento con: Metotrexate (MTX) 15 mg a settimana im o sc e Metilprednisone (MP) per os 2-4 mg/die (25 pazienti) oppure MTX 15 mg/settimana im o sc con MP 2-4 mg/die e colecalciferolo 300000 UI all'inizio dello studio (25 pazienti).

Dopo tre mesi di terapia le valutazioni basali saranno ripetute.

I risultati dello studio proposto aggiungeranno importanti informazioni sull'attività anti-infiammatoria e immunomodulatoria della 1,25-diidroossivitamina D3 supportandone un eventuale ruolo terapeutico nell'AR.

Background

L'artrite reumatoide (AR) è una malattia immunomediata, legata principalmente all'attivazione dei linfociti T helper (Th) di tipo 1, caratterizzata da infiammazione articolare e danno tissutale; a cui consegue una severa disabilità ed un'aumentata mortalità. Nell'AR il tessuto sinoviale è iperplasico ed induce la precoce comparsa d'erosione ossea con una progressiva distruzione delle articolazioni. L'erosione conduce a severe deformità delle articolazioni colpite ed ha un impatto negativo sulla qualità di vita (1).

Alterazioni del sistema immune nell'AR.

Le alterazioni del sistema immune nell'AR sono complesse e non ancora completamente conosciute. E' noto che un gran numero di linfociti T autoreattivi infiltra il tessuto sinoviale (2). Tali cellule esprimono marker d'attivazione dei Th1 memoria. che mediano la compartimentalizzazione dei T circolanti in risposta a molecole chemotattiche prodotte dalla sinovia. E' noto che le citochine secrete dai Th sono le principali responsabili del danno cellulo-mediato. Alcuni studi hanno evidenziato nell'AR una sostanziale differenza tra le citochine prodotte dai Th1 e quelle prodotte dai fibroblasti sinoviali. I linfociti Th1 di pazienti con AR sono in grado di produrre IFN γ, TNF, IL10 (3), e l'espressione d'IL2, IL4, IL5 e IL13 è bassa o assente, mentre nelle prime fasi di malattia, le cellule T della sinovia e le cellule stremali sono in grado di produrre IL2, IL4, IL13, IL17, IL15, il fattore di crescita per i fibroblasti e per le cellule epidermiche (4). Studi recenti hanno ipotizzato che questa differenza possa essere dovuta ad un difetto nel processo di differenziazione dei linfociti Th2, dovuto a varianti alleliche del recettore per IL 4. Questo conduce ad una minore attivazione di STAT e ad una minore produzione di proteina GATA-binding (5), entrambe richieste per la differenziazione del sottotipo linfocitario T helper 2.

La recente identificazione di una terza sottopopolazione di cellule Th, caratterizzata dalla produzione d'IL17A, IL17F (da qui deriva la denominazione di Th17) IL22, IL21 e TNF, ha fatto ipotizzare che proprio i linfociti Th17 potessero essere le cellule T implicate in alcune malattie autoimmuni (6). L'espressione d'IL17, IL17RA e IL17RC è stata osservata nel tessuto sinoviale ed osseo peri-articolare di pazienti con AR (7,8), mentre la produzione di IL17 è stata riscontrata nel fluido sinoviale e nel surnatante di culture di cellule mononucleate della sinovia (4,9). Il pattern citochinico nelle articolazioni (IL1 β, IL6 e in particolare IL23) sembrerebbe essere in grado di promuovere la differenziazione dei linfociti Th17. La distruzione del tessuto articolare e l'erosione ossea sono in parte mediate da linfociti T positivi per RANKL (recettore del fattore nucleare NF-KB) che induce la produzione di osteoclasti (10).

Per comprendere il meccanismo alla base delle malattie immuno-mediate è importante capire come si realizza la perdita della tolleranza periferica. Per questo molti studiosi hanno esaminato il numero e la funzione della sottopopolazione di Treg, per valutare se nell'infiammazione del tessuto sinoviale sia implicato un difetto nei meccanismi di tolleranza periferica. Gli studi condotti finora sono poco chiari perché, fino a poco tempo fa, la definizione di linfociti Treg era basata sull'espressione del CD4 e alti livelli dell'antigene CD25. Neanche l'inclusione del Foxp3 nel caratterizzare il fenotipo delle T reg ha risolto la questione poiché questo fattore di trascrizione, che svolge un ruolo fondamentale nei T reg, risulta essere espresso anche nei linfociti T CD4+ e CD25- (12). Permangono quindi dubbi sul ruolo dei linfociti T reg nell'AR: potrebbe essere importante lo studio della loro migrazione selettiva dal sangue periferico nel tessuto sinoviale e il loro conseguente accumulo nelle articolazioni (13).

La vitamina D e l'autoimmunità

L'1,25-diidrossi vitamina D, la forma attivata della vitamina D3, è un ormone steroideo con ruolo centrale nel metabolismo del calcio e nel rimodellamento osseo. La vitamina D esercita effetti pleiotropici sulla crescita e sul differenziamento di molti tipi cellulari, ed ha attività immunomodulante (14,18). Il recettore della vitamina D (VDR), oltre a regolare direttamente la funzione dei linfociti T e B, è in grado di influenzare il fenotipo e la funzione delle cellule dendritiche (DC); promuovendo la tolleranza ed una maggiore espressione di linfociti T reg anziché di quelli effettori (19). La capacità della vitamina D di influenzare positivamente la risposta immune è stata evidenziata da una importante e recente scoperta. In particolare è stato dimostrato che il calcitriolo inibisce la proliferazione dei linfociti T (20,21), ed in particolar modo del sottotipo Th 1 (22,23). L'aggiunta dell'1,25(OH)2D3 è in grado di diminuire la secrezione dell'IL2 dell'IFN γ da parte dei linfociti T CD4, mentre aumenta la produzione di IL5, 10 e del TGF β (24), con una maggiore risposta da parte della sottopopolazione linfocitaria Th2 (17). La produzione da parte dei linfociti Th17 dell'IL 17 che ha effetto pro-infiammatorio in diversi modelli sperimentali di autoimmunità organo specifica (cerebrale, cardiaco,tessuto sinoviale e intestinale) (25), è ridotta dall'aggiunta in coltura di l'1,25(OH)2D3. Questo avviene grazie alla diminuzione dei livelli d'IL6, che ha effetto di stimolo sui linfociti Th17.

Un recente studio sul modello animale, in vivo, ha dimostrato che il calcitriolo promuove l'attivazione dei linfociti Treg grazie ad uno specifico aumento delle citochine IL10, TGFβ e CTLA4 (24).

Il ruolo della vitamina D nella patogenesi e nel trattamento dell'AR è ancora controverso. In almeno 30.000 pazienti con RA un regolare introito di vitamina D ha ridotto il rischio di progressione della malattia (26): questo dato è stato confermato da un piccolo studio di coorte che ha dimostrato un'inversa correlazione tra concentrazione di vitamina D nel siero e la malattia in fase attiva. Tuttavia questo studio non ha riscontrato alcuna differenza nei livelli sierici di vitamina D tra i pazienti in studio e il gruppo di controllo (27). Due studi precedenti hanno esaminato i livelli di vitamina D in pazienti con AR conclamata: entrambi non mostrano alcuna correlazione tra la 25(OH)D ed i livelli di VES o PCR (28,29). I polimorfismi del VDR sono stati correlati con una maggiore suscettibilità all'AR (30,31). Si conosce ancora troppo poco il meccanismo con cui la vitamina D sarebbe in grado di modificare l'espressione delle malattie autoimmuni. Mahon e collaboratori hanno evidenziato che l'introduzione di 1000 IU/die di vitamina D e 800 mg di Calcio sono in grado di aumentare nel siero i livelli del TGF-b1 (fattore di crescita anti infiammatorio) (32). E' noto che l'aumento del TGF-b1 incrementa l'espressione di cellule dentritiche tollero gene e il numero di T reg; attenuando così la risposta del sistema immunitario (33,34).

Agonisti del VDR sono stati testati in due modelli di AR: l'artrite di Lyme e l'artrite collageno-indotta (35). L'1,25(OH)2D3 regola l'espressione delle metallo proteine di matrice e la produzione della prostaglandina E2 da parte dei fibroblasti della sinovia e dei condrociti articolari (36). Questo suggerisce che la vitamina D potrebbe avere un effetto immunomodulante sull'AR nell'uomo. Gli agonisti del VDR, quindi, dimostrano di poter essere utilizzati nel trattamento dell'AR, come suggerito dall'effetto benefico riscontrato in un trial open-label di 19 pazienti affetti da AR a cui si è somministrato alfacalcidiolo per 3 mesi (37). Nei modelli animali il trattamento con la vitamina 1,25(OH)2D3, nelle fasi precoci di malattia, ha prevenuto la progressione dell'artrite collageno-indotta (35). Con la somministrazione dell'1,25(OH)2D3 l'evoluzione dell'artrite risulta essere diminuita negli animali trattati rispetto ai controlli.

DESCRIZIONE DEL PROGRAMMA DI RICERCA

Esperimenti in vitro

Per verificare se i linfociti T di pazienti con AR sono ugualmente responsivi alla vitamina D rispetto ai controlli, paragonabili per sesso ed età, misureremo il VDR con la PCR ed il western blot nei PBMC frazionati (linfociti T, linfociti B e monociti) e non, sia in un gruppo di 10 nei pazienti con AR che in un gruppo di 10 soggetti di controllo.

Successivamente valuteremo, in 10 pazienti con AR e nel gruppo di controllo, la capacità dei PBMC frazionati e non d'idrossilare la 25OH vitamina D in posizione 1 per ottenere il metabolita attivo 1,25OH. Valuteremo anche la capacità di queste cellule di inattivare la vitamina D mediante un'idrossilazione in posizione 24.

I metaboliti saranno misurati con HPLC dopo un breve periodo d'incubazione con la 25OH vitamina D e l'1,25OH.

Questo studio ci consentirà di capire se i pazienti con AR hanno la stessa capacità, rispetto ai controlli sani di rispondere alla 1,25OH vitamina D e produrre l'ormone in forma attiva.

Esperimenti in vivo

Fase I

Arruoleremo nello studio 50 donne affette da AR in fase precoce di malattia, non precedentemente trattate con glucocorticoidi (GC) o farmaci modulanti l'attività di malattia (DMARDs).

I criteri d'inclusione prevedono l'arruolamento di donne maggiorenni con diagnosi d'AR, definita sulla base dei criteri formulati nel 1987 dal Collegio Americano dei Reumatologi (ACR; precedentemente denominata Associazione Americana delle malattie reumatiche) (38). Tali criteri diagnostici dovranno essere presenti da un periodo inferiore ai sei mesi prima dell'inclusione nello studio.

Saranno escluse dallo studio le pazienti che riceveranno diagnosi di un altro tipo d'artrite infiammatoria o artrite secondaria non su base infiammatoria. Le pazienti con storia di tubercolosi o con segni radiografici di malattia polmonare attiva con storia di cancro; malattia renale severa/non controllata o in progressione, malattia epatica, ematologica, gastrointestinale, polmonare, cardiaca,

neurologica o cerebrale saranno escluse. Infine saranno escluse le pazienti che hanno ricevuto da meno di 6 mesi una terapia biologica (o Etanercept e/o Anakinra entro 3 mesi).

Sarà inoltre arruolato un gruppo di controllo costituito da donne sane paragonabili per età, le donne saranno raccolte tra quelle spontaneamente afferenti al centro di Malattie Metaboliche dell'Osso diretto dal Prof. Isaia e risultate normali all'esame densitometrico (T score maggiore di -1,0 SD secondo i criteri del WHO). Saranno escluse dallo studio pazienti affette da patologie o che assumano farmaci in grado di alterare i livelli di 25 OH vitamina D, il profilo immunologico o l'osteoclastogenesi.

Valutazione iniziale delle pazienti

In tutte le pazienti misureremo il livello basale di 25 OH vitamina D nel siero e sarà somministrato un questionario sull'introito di vitamina D con la dieta, tramite software PROGEO Photo Intake.

Valutazioni cliniche

-Grado di progressione dell'AR: valutata mediante un punteggio che si basa sul grado di attività di malattia (DAS28) (39).

-L'intensità del dolore verrà valutata con la Scala Analogica Visiva (VAS).

-Perdita di funzionalità: la disabilità funzionale verrà valutata col questionario Health Assessment in cui al grado 0 corrisponde l'assenza di limitazioni, mentre al grado 3 corrisponde una severa perdita di funzione. Sarà inoltre somministrato alle pazienti un questionario, con una scala da 0 a 100, sulle condizioni generali di salute (40).

In tutti i soggetti misureremo il livello basale di 25 OH vitamina D nel siero, l'intake di vitamin D con la dieta, la VES (velocità d'eritrosedimentazione) e la PCR (proteina C reattiva).

Isolamento e messa in cultura delle cellule

Le cellule mononucleate da sangue periferico (PBMC) saranno ottenute in tutte le pazienti con il metodo Ficoll-Paque da 40 ml di sangue periferico in litio eparina, come in precedenza descritto (41).

Citofluorimetria

Per quantificare i precursori degli osteoclasti e le sottopolazioni di linfociti T nel sangue periferico utilizzeremo la tecnica citofluorimetrica.

E’ stato osservato che i precursori degli osteoclasti circolanti sono compresi all’interno della frazione dei monociti del sangue periferico: essi costituiscono un pool di rifornimento di pre-osteoclasti per il midollo osseo e possono essere richiamati nell’osso e nelle articolazioni a seguito di processi riparativi o patologici. In particolare si pensa che questi precursori siano reclutati nel processo di rimodellamento osseo fisiologico e durante il processo di riparazione delle fratture, in processi patologici essi sono prodotti in eccesso. Ciò si traduce in una significativa perdita di massa ossea, condizione che si riscontra in numerose situazioni che alterano l’omeostasi dello scheletro come nelle metastasi ossee (42), nel mieloma multiplo (43) e nell’osteoporosi postmenopausale (41).

I precursori degli osteoclasti saranno identificati marcando i campioni di sangue fresco con anticorpi anti VNR, anti CD14 e anti CD11-b, o con l’istotipo ed incubati per 30 minuti ad una temperatura di 4°C. Le cellule triple positive (CD14+/CD11b+ / VNR+)saranno considerate, in accordo con la letteratura, precursori degli osteoclasti (41).

Per identificare la sottopopolazione di linfociti T marcheremo le cellule con anticorpi anti CD3, anti CD4, anti CD8, anti CD25 e anti FOXP3. L’analisi al FACS misurerà i linfociti CD3+/CD4+, i CD3+/CD8+ e le cellule CD3+/CD4+/CD25+/FOXP3+ (T reg).

La citofluorimetria verrà effettuata con il FACS (Becton Dickinson e Co).

Misurazione delle citochine

Per identificare le differenti sottopopolazioni di linfociti T sarà utilizzata la tecnica ELISPOT per misurare l’ INFγ ed ilTNF (Th1), l’IL 4 (Th2), l’IL17 (Th17) e il TGFβ (T reg). Per valutare la produzione di citochine dei PBMC in condizioni simili a ciò che avviene in vivo, senza le possibili alterazioni legate alla messa in cultura, e per individuare i tipi cellulari coinvolti, noi confronteremo i PBMC al basale e dopo stimolo per 24 ore con fitoemoagglutinina (PHA), un mitogeno che stimola principalmente i linfociti T.

I PBMC isolati a fresco ($1*10^6$/pozzetto) saranno messi in cultura su piastre da 96 pozzetti con RPMI supplementato con siero bovino fetale (FBS) al 10%, benzil penicillina (100 UI/ml) e streptomicina (100 µg/ml), cono senza l’aggiunta PHA (10 µg/ml).

Per valutare il ruolo dei linfociti T nel metabolismo osseo andremo anche a misurare, mediante la tecnica ELISA, i livelli di RANKL e d’OPG prodotti dai PBMC in coltura. Queste citochine saranno messe in relazione con l’attività osteoclastogenica dei PBMC, con le diverse sottopopolazioni di linfociti T e con i livelli di 25OH vitamina D.

Questi esperimenti ci consentiranno di identificare i PBMC responsabili della produzione di citochine nei pazienti con AR al basale e dopo terapia. Si utilizzerà la PHA per valutare la possibile alterazione, nei pazienti con AR, nella risposta immunitaria dei linfociti T stimolati.

Real time PCR

Per meglio caratterizzare le differenti sottopopolazioni di linfociti T misureremo con la real time PCR i markers d'attivazione dei Th1/Th2/Treg/Th17: in particolar modo misureremo T-bet e STAT-4 (Th1), GATA-3, STAT-6 (Th2), RORγT (TH17) e FOXP3 (Treg).

I risultati ottenuti saranno confrontati con quelli già presenti in letteratura,ricercando una possibile correlazione tra linfociti T, livelli basali di 25OH vitamina D e osteoclasto- genesi.

Seconda parte dello studio

Dopo le valutazioni basali, nella seconda parte dello studio, le pazienti saranno randomizzate in doppio cieco ad uno specifico gruppo di trattamento con:

- Metotrexate (MTX) 15 mg a settimana im o sc e Metilprednisone (MP) per os 2-4 mg/die e placebo all'inizio dello studio (25 pazienti)

oppure

- MTX 15 mg/settimana, im o sc, con MP 2-4 mg/die e colecalciferolo 300000 UI per os all'inizio dello studio (25 pazienti).

Dopo tre mesi di terapia saranno ripetute le valutazioni basali.

Analisi statistica.

Le statistiche saranno eseguite con il softaware SPSS 18.0.

Per paragonare la risposta alla vitamina D in vitro nei vari tipi cellulari ottenuti da pazienti affette da AR o da controlli verrà impiegato il T test di Student per dati non-appaiati

Pazienti al basale e controlli verranno confrontati per le variabili analizzate con l'ANOVA ad una via, questo test ci permetterà sia di escludere bias di selezione della casistica sia di evidenziare le caratteristiche differenti tra i gruppi

Verrà costruito un modello di regressione multilineare per valutare la relazione tra sottotipi linfocitari, citochine, pre-osteoclasti circolanti, status vitaminico D come covariata utilizzeremo

l'età e come fattore fisso la presenza di AR. Per le pazienti affette da AR nel modello verrà inserita l'attività di malattia (VAS, DAS28, qualità di vita, VES e PCR).

Per valutare l'effetto della terapia con o senza colecalciferolo sul fenotipo linfocitario, sugli osteoclasti circolanti, sulla produzione di citochine e sull'attività di malattia (VAS, DAS28, qualità di vita, VES e PCR) sarà applicato il T test di Student per dati appaiati.

COMPITI DEL CENTRO COORDINATORE

1. la SS Reumatologia dell'ASO Ordine Mauriziano di Torino sarà il centro coordinatore
2. recluterà le pazienti affette da AR in fase precoce secondo i criteri d'inclusione sopra citati
3. somministrerà farmaco e placebo
4. rileverà i parametri di attività di malattia secondo l'iter gestionale standard dell'artrite reumatoide (VES, PCR,VAS, DAS 28, GH)

COMPITI DEL CENTRO SPERIMENTATORE

5. l'unità del Prof. Isaia presso l'Ospedale Molinette di Torino sarà il centro sperimentatore
6. recluterà i soggetti di controllo tra soggetti sani afferenti al servizio di Densitometria ossea dell'Ospedale Molinette, risultati negativi all'esame.
7. eseguirà gli esperimenti in vitro per testare la capacità dei PBMC e dei linfociti T di pazienti affette da AR di rispondere alla somministrazione di vitamina D in coltura e di metabolizzarla attivandola od inattivandola
8. eseguirà gli esperimenti al basale (50 pazienti e 50 controlli) e dopo 3 mesi di terapia su PBMC delle pazienti (50) con tecniche FACS, ELISPOT, real time PCR
9. eseguirà i dosaggi delle citochine infiammatorie prodotte dalle PBMC dei soggetti al basale e delle pazienti dopo terapia.
10. eseguirà la valutazione dell'apporto alimentare di vitamina d tramite il software Progeo Photo Intake.
11. eseguirà il dosaggio della 25 OH Vitamina D al basale
12. eseguirà le analisi statistiche conclusive con software SPSS 15.0
