# Supplementary material for: Vitamin D and immunomodulation in early rheumatoid arthritis: A randomized double-blind placebo-controlled study
Source: PLoS One. 2017 Jun 5;12(6):e0178463. doi: 10.1371/journal.pone.0178463 (PMC5459341; doi:10.1371/journal.pone.0178463)
Supplement: S3 File — Protocol of the study approved by the Ethical committee in English language. (PDF) [file pone.0178463.s003.pdf]

VITAMIN D EFFECT ON T LYMPHOCYTES AND OSTEOCLASTOGENESIS IN RHEUMAOID ARTHRITIS

Abstract (5000)

Rheumatoid arthritis (RA) is an immune-mediated disease characterized by articular inflammation and subsequent tissue damage, which lead to severe disability and increased mortality. RA is characterized by invasive synovial hyperplasia leading to progressive joint destruction. Radiographic studies have shown that bone erosion in RA begins at an early stage of the disease and gradually or rapidly exacerbates. Bone erosion results in severe deformities of the affected joints and impairs normal activity and quality of life.

The immune alterations in RA are complex and not completely understood, it is generally accepted that T helper (Th) 1 cells are the main responsible for the immune alteration together with an increase in Th17 and a decrease in T regulatory cells (T reg). Furthermore there is an increase in osteoclasts formation and activity both at the level of the inflamed joint both at systemic level. Recent in vitro and in vivo data suggest that 1,25-Dihydroxyvitamin D3 [1,25(OH)2D3], the bioactive form of vitamin D3, is exerts pleiotropic effects on the growth and differentiation of many cell types, and displays exquisite immunoregulatory properties. It has been suggested that [1,25(OH)2D3] exerts direct modulatory effects on T-cell and B-cell function and on dendritic cells (DCs), thereby promoting tolerogenic properties that favor the induction of regulatory, rather than effector, T cells.

The role of vitamin D in the pathogenesis and in the treatment of RA is still controversial. Some studies suggested a role for low vitamin D intake in the pathogenesis of RA and others also suggest a potential therapeutic effect for vitamin D in RA.

The aim of this study are to:

1- explore the role of vitamin D in the pathogenesis of early RA

2- evaluate the role of vitamin D as immunomodulator in early RA
3- evaluate the effect of vitamin D on osteoclastogenesis in early RA
4- evaluate the clinical effect of vitamin D in early RA

In vitro experiments

In order to test if T cells from RA patients are equally responsive to vitamin D as respect to healthy age and sex matched controls we will measure vitamin D receptor (VDR) by real time PCR and western blot in fractoned (T cells, B cells and monocytes) and unfractioned PBMC from RA patients and controls.

To evaluate if PBMC from RA patients and healthy donors has the same ability to produce and /or inactivate vitamin D we will explore the ability of fractioned and unfractioned PBMC from RA patients and healthy donors to hydroxilate 25 OH vitamin D in position 1 to obtain the active metabolite 1,25 OH vitamin D, and the ability of these cells to inactivate vitamin D through hydroxylation in position 24. The metabolites will be measure after a short incubation with 25 OH vitamin d and 1-25OH vitamin D by HPLC.

In vivo experiments

We will enrol in the study 50 female patients affected by early phase RA not previously treated wit glucocorticoid or disease modifying activity drugs, and 50 healthy controls age matched. At baseline in all the subjects we will measure serum 25 OH vitamin D, a questionnaire on vitamin D dietary intake will be distributed to each patient.

In order to quantify osteoclast precursors and T cells subsets in PBMC we will use flow cytometry. To further characterise the T helper phenotype we will measure the cytokine profile expression and the markers of Th1/Th2/Treg/Th17 by real time PCR.

To assess cytokines production by PBMC and to detect the cells involved, we will compare PBMC at baseline and after stimulation with phytoemoagglutinin, a mitogen that predominantly stimulates T cells, we will use ELISA and ELISPOT techniques.

The measurement of T cells subset will be correlated with the other data and will suggest a possible relation between T cells, basal levels of 25OH vitamin D and osteoclastogenesis.

In order to evaluate the role of T cells in bone metabolism we will also measure the levels of RANKL and OPG in PBMC cultures. These cytokines will be correlated with osteoclastogenesis, T cells subset and 25OH vitamin D levels. Furthermore we will compare patients and controls for the analysed parameters.

After the baseline evaluation the patients will be included in the second part of the study and randomly assigned to one treatment group that will consist in:

methotrexate (MTX) 15 mg/week im or sc and metilprednisolone (MP) per os 2-4 mg/day (25 patients) or MTX 15 mg/week plus MP 2-4 mg/day plus cholecalciferol 300000 UI at the beginning of the study (25 patients). After three months of therapy the baseline evaluations will be repeated. The results of the proposed study will add important notions to the complex immunomodulatory and anti-inflammatory activity of 1,25-dihydroxyvitamin D3 and might support its therapeutic role in RA.

BACKGROUND (8000).

Rheumatoid arthritis (RA) is an immune-mediated disease mainly driven by Th1 cells characterized by articular inflammation and subsequent tissue damage, which lead to severe disability and increased mortality. RA is characterized by invasive synovial hyperplasia leading to progressive joint destruction. Radiographic studies have shown that bone erosion in RA begins at an early stage of the disease and gradually or rapidly exacerbates. Bone erosion results in severe deformities of the affected joints and impairs normal activity and quality of life [1].

Immune alteration in RA

The immune alterations in RA are complex and not completely understood. A high number of auto reactive T cells infiltrate the synovium [2]: synovial T cells express an extensive array of cell surface antigens that reflect prior antigen experience. Principal among these are markers of T helper 1 (Th1) memory cells, including the expression of specific chemokine receptors and integrins. The profile of chemokine receptors suggests that there is an element of selective trafficking of T-cell subsets in response to local production of chemoattractants by synovial stroma. By convention, it is acknowledged that cytokines secreted by lineage specific Th cells provide the ‘flavor’ of the effector phase of cell-mediated immunity. In RA many studies have provided evidence for a Th1 bias, with the cytokine profile expressed by synovial fibroblast. Th1 cells derived from patients with established disease markedly expresses IFN-γ and TNF, but also IL-10 [3]. Expression of IL-2, IL-4, IL-5, and IL-13 is low or absent. This contrasts with the profile of cytokines expressed by synovial T cells and stromal cells very early in disease, which is dominated by IL-2, IL-4, IL-13, IL-17, IL-15, basic fibroblast growth factor, and epidermal growth factor [4]. The mechanisms underlying this transition are unclear, although reported data have led to the proposal that there is an intrinsic defect in Th2 differentiation that is explained in part by allelic variants of the IL-4R, which attenuate activation of STAT6 and induction of

GATA-binding protein 3 [5], both of which are requisites for commitment to Th2 cell lineage differentiation. The recent excitement following the identification of a third Th cell subset, characterized by the expression of IL-17A and IL- 17F (hence termed Th17), as well as IL-22, IL-21 and TNF, has raised the possibility that Th17 cells may be an important effector T-cell subset in diseases such as RA, multiple sclerosis, and inflammatory bowel disease [6]. Expression of IL-17, IL-17RA, and IL-17RC has been described in RA synovial tissue and juxta-articular bone by immunohistochemistry [7, 8], and IL-17 protein production has been detected in synovial fluid and culture supernatants of synovial mononuclear cell explants [4, 9]. Indeed, the cytokine milieu in the joint, with IL-1β, IL-6, and IL-23 in particular, would certainly support Th17 differentiation. Joint and bone matrix destruction is also induced in part through RANKL (receptor activator for nuclear factor-κB ligand)-positive T cells that promote bone erosion through activation and differentiation of osteoclast precursors [10]. A major challenge to our understanding of the pathogenesis of immune-mediated diseases is to elucidate why pathways of peripheral tolerance might fail. To this end, several authors investigated the number and function of regulatory cell subsets, in order to test the hypothesis that at sites of synovial inflammation there may be defects in pathways of peripheral tolerance. Studies have been thwarted to an extent because until recently the enumeration of subsets of naturally occurring regulatory T cells was based purely on CD4 and non selective high level expression of the T-cell activation antigen CD25 (hereafter referred to as regulatory T cells [Treg]). Even the inclusion of Foxp3 into the phenotyping armamentarium has not solved the problem, because this master transcription factor, which plays a central role in the function of Treg [11], is also induced upon TCR engagement of CD4+CD25- T cells [12]. Furthermore, there remains controversy as to whether there are genuine defects in the numbers of circulating Tregs in RA. This may have as much to do with their selective migration and accumulation in synovial joints compared with peripheral blood [13],

Vitamin D and autoimmune diseases.

1,25-Dihydroxyvitamin D3 [1,25(OH)2D3], the bioactive form of vitamin D3, is a secosteroid hormone that has a central function in calcium and bone metabolism. It also exerts pleiotropic effects on the growth and differentiation of many cell types, and displays exquisite immunoregulatory properties [14-18].

In addition to exerting direct modulatory effects on T-cell and B-cell function, vitamin D receptor (VDR) agonists influence the phenotype and function of dendritic cells (DCs), thereby promoting tolerogenic properties that favour the induction of regulatory, rather than effector, T cells [19]. The capability of the vitamin D system to enhance innate immune responses has been highlighted by important, novel findings. In particular it has been demonstrated that calcitriol inhibits T-lymphocyte proliferation [20, 21]; particularly of the Th1 arm [22, 23]. Addition of 1,25(OH)2D3 leads to decreased secretion of IL-2 and IFN-γ by CD4 T cells and promotes IL-5, IL-10 and TGF β [24] production, which further shift the T cell response towards Th2 dominance [17]. T production of IL-17, a proinflammatory cytokine that is produced by pathogenic T cells (Th17) in various models of organ-specific autoimmunity in the brain, heart, synovium and intestines [25] is reduced by the addition of 1,25(OH)2D3 trough the reduction of IL-6, an important factor that stimulates Th17 cells. A recent in vivo study on animal model shows that calcitriol promotes T regulatory (T reg) functions with distinct increases of the regulatory set of IL-10, TGFβ, FoxP3, and CTLA4 [24]. The role of vitamin D in the pathogenesis and in the treatment of RA is still controversial. In almost 30,000 patients with RA the regular vitamin D intake reduced the risk of developing the disease [26], this datum was confirmed by a smallest cohort study that show an inverse correlation between serum vitamin D and disease activity, but does not found any difference between serum levels of patients an controls [27]. Two previous studies have examined vitamin D levels in patients with established RA. Both showed no

relationship between 25(OH)D and C reactive protein or eritrocytes sedimentation rate [28, 29].

Also the polymorphisms in the VDR have been correlated with increased susceptibility of RA [30, 31]. Yet, little is known about how vitamin D modifies the development of autoimmune diseases. Mahon et al. described that 1000 IU/day vitamin D and 800 mg Ca intake increased the anti-inflammatory transforming growth factor (TGF)-b1 serum [32]. It is well known that the increase in TGF-b1 induce tolerogenic dendritic cells and T reg that down regulates the immune response [33, 34]. VDR agonists have been tested in two RA models, namely Lyme arthritis and collagen-induced arthritis [35]. 1,25(OH)2D3 contributes to the regulation of matrix metalloproteinase and prostaglandin E2 production by human rheumatoid synovial fibroblasts and articular chondrocytes [36] which suggests it could also have immunomodulatory effects in human RA. VDR agonists indeed show potential as a treatment for RA as indicated by the beneficial effects of administration of alphacalcidiol in a 3-month, open-label trial of 19 patients with RA [37] In mice models 1,25(OH)2D3 vitamin treatment in the early phase, of collagen-induced arthritis was preventable to a certain extent [35]. With the administration of 1,25(OH)2D3 the progression of arthritis decreased compared with the untreated control animals.

REFERENCES

[1] Goldring SR Pathogenesis of bone erosions in rheumatoid arthritis. Curr Opin Rheumatol 2002;14:406-10.

[2] Duke O, Panayi GS, Janossy G, Poulter LW An immunohistological analysis of lymphocyte subpopulations and their microenvironment in the synovial membranes of patients with rheumatoid arthritis using monoclonal antibodies. Clin Exp Immunol 1982;49:22-30.

[3] McInnes IB, Schett G Cytokines in the pathogenesis of rheumatoid arthritis. Nat Rev Immunol 2007;7:429-42.

[4] Raza K, Scheel-Toellner D, Lee CY, Pilling D, Curnow SJ, Falciani F, Trevino V, Kumar K, Assi LK, Lord JM, Gordon C, Buckley CD, Salmon M Synovial fluid leukocyte apoptosis is inhibited in patients with very early rheumatoid arthritis. Arthritis Res Ther 2006;8:R120.

[5] Prots I, Skapenko A, Wendler J, Mattyasovszky S, Yone CL, Spriewald B, Burkhardt H, Rau R, Kalden JR, Lipsky PE, Schulze-Koops H Association of the IL4R single-nucleotide polymorphism I50V with rapidly erosive rheumatoid arthritis. Arthritis Rheum 2006;54:1491-500.

[6] Weaver CT, Hatton RD, Mangan PR, Harrington LE IL-17 family cytokines and the expanding diversity of effector T cell lineages. Annu Rev Immunol 2007;25:821-52.

[7] Chabaud M, Durand JM, Buchs N, Fossiez F, Page G, Frappart L, Miossec P Human interleukin-17: A T cell-derived proinflammatory cytokine produced by the rheumatoid synovium. Arthritis Rheum 1999;42:963-70.

[8] Zrioual S, Toh ML, Tournadre A, Zhou Y, Cazalis MA, Pachot A, Miossec V, Miossec P IL-17RA and IL-17RC receptors are essential for IL-17A-induced ELR+ CXC chemokine expression in synoviocytes and are overexpressed in rheumatoid blood. J Immunol 2008;180:655-63.

[9] Chabaud M, Garnero P, Dayer JM, Guerne PA, Fossiez F, Miossec P Contribution of interleukin 17 to synovium matrix destruction in rheumatoid arthritis. Cytokine 2000;12:1092-9.

[10] Udagawa N, Kotake S, Kamatani N, Takahashi N, Suda T The molecular mechanism of osteoclastogenesis in rheumatoid arthritis. Arthritis Res 2002;4:281-9.

[11] Zheng Y, Rudensky AY Foxp3 in control of the regulatory T cell lineage. Nat Immunol 2007;8:457-62.

[12] Wang J, Ioan-Facsinay A, van der Voort EI, Huizinga TW, Toes RE Transient expression of FOXP3 in human activated nonregulatory CD4+ T cells. Eur J Immunol 2007;37:129-38.

[13] Cao D, van Vollenhoven R, Klareskog L, Trollmo C, Malmstrom V CD25brightCD4+ regulatory T cells are enriched in inflamed joints of patients with chronic rheumatic disease. Arthritis Res Ther 2004;6:R335-46.

[14] Deluca HF, Cantorna MT Vitamin D: its role and uses in immunology. Faseb J 2001;15:2579-85.

[15] Griffin MD, Xing N, Kumar R Vitamin D and its analogs as regulators of immune activation and antigen presentation. Annu Rev Nutr 2003;23:117-45.

[16] Adorini L Intervention in autoimmunity: the potential of vitamin D receptor agonists. Cell Immunol 2005;233:115-24.

[17] van Etten E, Mathieu C Immunoregulation by 1,25-dihydroxyvitamin D3: basic concepts. J Steroid Biochem Mol Biol 2005;97:93-101.

[18] Arnson Y, Amital H, Shoenfeld Y Vitamin D and autoimmunity: new aetiological and therapeutic considerations. Ann Rheum Dis 2007;66:1137-42.

[19] Adorini L, Giarratana N, Penna G Pharmacological induction of tolerogenic dendritic cells and regulatory T cells. Semin Immunol 2004;16:127-34.

[20] Bhalla AK, Amento EP, Serog B, Glimcher LH 1,25-Dihydroxyvitamin D3 inhibits antigen-induced T cell activation. J Immunol 1984;133:1748-54.
[21] Lemire JM Immunomodulatory role of 1,25-dihydroxyvitamin D3. J Cell Biochem 1992;49:26-31.
[22] Mattner F, Smiroldo S, Galbiati F, Muller M, Di Lucia P, Poliani PL, Martino G, Panina-Bordignon P, Adorini L Inhibition of Th1 development and treatment of chronic-relapsing experimental allergic encephalomyelitis by a non-hypercalcemic analogue of 1,25-dihydroxyvitamin D(3). Eur J Immunol 2000;30:498-508.
[23] Boonstra A, Barrat FJ, Crain C, Heath VL, Savelkoul HF, O'Garra A 1alpha,25-Dihydroxyvitamin d3 has a direct effect on naive CD4(+) T cells to enhance the development of Th2 cells. J Immunol 2001;167:4974-80.
[24] Daniel C, Sartory NA, Zahn N, Radeke HH, Stein JM Immune modulatory treatment of trinitrobenzene sulfonic acid colitis with calcitriol is associated with a change of a T helper (Th) 1/Th17 to a Th2 and regulatory T cell profile. J Pharmacol Exp Ther 2008;324:23-33.
[25] Steinman L A brief history of T(H)17, the first major revision in the T(H)1/T(H)2 hypothesis of T cell-mediated tissue damage. Nat Med 2007;13:139-45.
[26] Merlino LA, Curtis J, Mikuls TR, Cerhan JR, Criswell LA, Saag KG Vitamin D intake is inversely associated with rheumatoid arthritis: results from the Iowa Women's Health Study. Arthritis Rheum 2004;50:72-7.
[27] Cutolo M, Otsa K, Laas K, Yprus M, Lehtme R, Secchi ME, Sulli A, Paolino S, Seriolo B Circannual vitamin d serum levels and disease activity in rheumatoid arthritis: Northern versus Southern Europe. Clin Exp Rheumatol 2006;24:702-4.
[28] Oelzner P, Muller A, Deschner F, Huller M, Abendroth K, Hein G, Stein G Relationship between disease activity and serum levels of vitamin D metabolites and PTH in rheumatoid arthritis. Calcif Tissue Int 1998;62:193-8.
[29] Kroger H, Penttila IM, Alhava EM Low serum vitamin D metabolites in women with rheumatoid arthritis. Scand J Rheumatol 1993;22:172-7.
[30] Garcia-Lozano JR, Gonzalez-Escribano MF, Valenzuela A, Garcia A, Nunez-Roldan A Association of vitamin D receptor genotypes with early onset rheumatoid arthritis. Eur J Immunogenet 2001;28:89-93.
[31] Maalej A, Petit-Teixeira E, Michou L, Rebai A, Cornelis F, Ayadi H Association study of VDR gene with rheumatoid arthritis in the French population. Genes Immun 2005;6:707-11.
[32] Mahon BD, Gordon SA, Cruz J, Cosman F, Cantorna MT Cytokine profile in patients with multiple sclerosis following vitamin D supplementation. J Neuroimmunol 2003;134:128-32.
[33] Adorini L, Penna G, Giarratana N, Roncari A, Amuchastegui S, Daniel KC, Uskokovic M Dendritic cells as key targets for immunomodulation by Vitamin D receptor ligands. J Steroid Biochem Mol Biol 2004;89-90:437-41.
[34] Cantorna MT, Woodward WD, Hayes CE, DeLuca HF 1,25-dihydroxyvitamin D3 is a positive regulator for the two anti-encephalitogenic cytokines TGF-beta 1 and IL-4. J Immunol 1998;160:5314-9.
[35] Cantorna MT, Hayes CE, DeLuca HF 1,25-Dihydroxycholecalciferol inhibits the progression of arthritis in murine models of human arthritis. J Nutr 1998;128:68-72.
[36] Tetlow LC, Woolley DE Effects of 1 alpha,25dihydroxyvitaminD3 on matrix metalloproteinase expression by rheumatoid synovial cells and articular chondrocytes in vitro. Ann N Y Acad Sci 1999;878:615-8.
[37] Andjelkovic Z, Vojinovic J, Pejnovic N, Popovic M, Dujic A, Mitrovic D, Pavlica L, Stefanovic D Disease modifying and immunomodulatory effects of high dose 1 alpha (OH) D3 in rheumatoid arthritis patients. Clin Exp Rheumatol 1999;17:453-6.
[38] Arnett FC, Edworthy SM, Bloch DA, McShane DJ, Fries JF, Cooper NS, Healey LA, Kaplan SR, Liang MH, Luthra HS, et al. The American Rheumatism Association 1987

revised criteria for the classification of rheumatoid arthritis. Arthritis Rheum 1988;31:315-24.
[39] Prevoo ML, van 't Hof MA, Kuper HH, van Leeuwen MA, van de Putte LB, van Riel PL Modified disease activity scores that include twenty-eight-joint counts. Development and validation in a prospective longitudinal study of patients with rheumatoid arthritis. Arthritis Rheum 1995;38:44-8.
[40] Palmer D, El Gaafary M, El Miedany Y Improving patient care: measurement of outcome in rheumatoid arthritis. Br J Nurs 2007;16:1010-5.
[41] D'Amelio P, Grimaldi A, Di Bella S, Brianza SZ, Cristofaro MA, Tamone C, Giribaldi G, Ulliers D, Pescarmona GP, Isaia G Estrogen deficiency increases osteoclastogenesis up-regulating T cells activity: a key mechanism in osteoporosis. Bone 2008;43:92-100.
[42] Roato I, D'Amelio P, Gorassini E, Grimaldi A, Bonello L, Fiori C, Delsedime L, Tizzani A, De Libero A, Isaia G, Ferracini R Osteoclasts are active in bone forming metastases of prostate cancer patients. PLoS ONE 2008;3:e3627.
[43] Brunetti G, Colucci S, Pignataro P, Coricciati M, Mori G, Cirulli N, Zallone A, Grassi FR, Grano M T cells support osteoclastogenesis in an in vitro model derived from human periodontitis patients. J Periodontol 2005;76:1675-80.

Research Program Description and Research Unit Duties (16000)

In vitro experiments

In order to test if T cells from RA patients are equally responsive to vitamin D as respect to healthy age and sex matched controls we will measure VDR by real time PCR and western blot in fractioned (T cells, B cells and monocytes) and unfractioned PBMC from 10 RA patients and 10 controls.

Furthermore we will explore the ability of fractioned and unfractioned PBMC from RA patients and healthy donors to hydroxilate 25 OH vitamin D in position 1 to obtain the active metabolite 1,25 OH vitamin D. We will also explore the ability of these cells to inactivate vitamin D through hydroxylation in position 24. The metabolites will be measure after a short incubation with 25 OH vitamin D and 1-25OH vitamin D by HPLC.

This experiments will allow us to define if RA patients have the same ability of healthy donors to answer to 1-25OH vitamin D and to produce their own hormone.

In vivo experiments

PHASE I

We will enrol in the study 50 female patients affected by early phase RA not previously treated wit glucocorticoid (GC) or disease modifying activity drugs (DMARDs).

Eligible patients will be female ≥18 years of age with a diagnosis of RA, as defined by the American College of Rheumatology (ACR; formerly, the American Rheumatism Association) 1987 [38] criteria for < 6 months prior to inclusion in the study.

Exclusion criteria consist of diagnoses of any other inflammatory arthritis or a secondary non-inflammatory arthritis. Patients with a history of tuberculosis or a chest radiograph showing active tuberculosis will be also excluded.

Patients who had a history of malignancy, or severe, progressive, and/or uncontrolled renal, hepatic, hematologic, gastrointestinal, endocrine, pulmonary, cardiac, neurologic, or cerebral disease will be excluded. Patients who had received any biologic therapy within 6 months (or had received etanercept and/or anakinra within 3 months) will be excluded.

50 healthy control women will be enrolled in the bone disease centre directed by Prof Isaia. The women have to be normal at densitometric test (T-score higher than -1.0 SD according to the WHO criteria). Patients with diseases or taking drugs active on vitamin D metabolism or on immune system will be excluded

Baseline evaluation

At baseline in all the subjects we will measure serum 25 OH vitamin D, a questionnaire on vitamin D dietary intake will be distributed to each patient.

Clinical evaluation of RA patients

Disease activity: The rheumatic disease activity will be measured by disease activity score 28 (DAS 28) [39]. The pain will be measured with the Visual Analogical Scale (VAS)

Disability: The physical disability will be evaluated by the Health Assessment Questionnaire where zero implies no handicap and three severe disabilities and with the general health questionnaire evaluated by the patients with a scale from 0 to 100 [40].

The erythrocyte sedimentation rate (ESR) and C-reactive protein (CRP) will be measured in all the patients.

Cell isolation and cultures

Peripheral blood mononuclear cells (PBMC) will be obtained with the Ficoll-Paque method from 40 ml peripheral blood in lithium heparin as previously described [41].

Flow cytometry

In order to quantify osteoclast precursors and T cells subset in peripheral blood we will use flow cytometry. It has been demonstrated that circulating osteoclast precursors exist primarily within the monocytic fraction of peripheral blood, and their presence in the circulation serves both as a reservoir for replenishing pre-osteoclast populations in the bone marrow as needed and as a potentially abundant source of pre-osteoclasts that can be recruited into bone or joint tissue in response to reparative or pathological signals. In contrast to the beneficial nature of pre-osteoclast recruitment during normal bone remodelling or fracture repair, excessive pre-osteoclast recruitment in pathological conditions causes significant bone loss in many skeletal disorders characterized by increased osteoclast formation and activity as bone metastases [42], in postmenopausal osteoporosis [41] or multiple myeloma [43].

Osteoclast precursors will be detected by staining the fresh blood samples with FITC-conjugated anti-VNR, PE-conjugated anti CD14 and APC-conjugated anti-CD11b mAb, or with the corresponding isotype control followed by incubation at 4°C for 30 min. The triple-positive (CD14+/CD11b+/VNR+) cells will be considered as osteoclast precursors according to previous literature [41].

To evaluate T-cells subset we will stain the cells with anti-CD3, anti CD4, anti CD8, anti CD 25 and anti FOXP3 antibodies. The FACS analyses will allow us to measure CD3+/CD4+ cells as respect to CD3+/CD8+ cells and CD3+/CD4+/CD25+/FOXP3+ (T reg) cells.

Flow cytometry will be performed on a FACSCalibur flow cytometer (Becton Dickinson & Co.).

Cytokines measurement.

In order to identify different T cell subsets we will use ELISPOT technique for IFNγ and TNF (Th1), IL4 (Th2), IL17 (Th17) and TGFβ (Treg). We will assess the cytokines production by PBMC in conditions similar to those in vivo free from the possibility of alteration due to residence in culture, and to detect the cells involved, we will compare PBMC at baseline and after stimulation for 24 hours with phytoemoagglutinin (PHA), a mitogen that predominantly stimulates T cells. Freshly isolated PBMC ($1x10^6$ PBMC/well) will be plated in triplicate in 96-well plates using RPMI supplemented with 10% fetal bovine serum (FBS), benzyl penicillin (100 IU/ml) and streptomycin (100 μg/ml), designated complete medium, or in complete medium plus PHA [10 μg/ml].

In order to evaluate the role of T cells in bone metabolism we will also measure the levels of RANKL and OPG in PBMC cultures by ELISA tecnique. These cytokines will be correlated with osteoclastogenesis, T cells subset and 25OH vitamin D levels.

These experiments will allow us to find out which of the PBMC produce the higher amount of cytokines in RA patients at baseline and after therapy. The use of PHA will allow us to evaluate a possible alteration in the answer to immune stimulation in T cells from RA patients.

Real time PCR.

To further characterise the T helper phenotype we will measure the markers of Th1/Th2/Treg/Th17 by real time PCR: in particular we will measure T-bet and STAT-4 (Th1), GATA-3, STAT-6 (Th2), RORγT (TH17) and FOXP3 (Treg).

The measurement of T cells subsets and cytokines production will be correlated with the other data and will suggest a possible relation between T cells, basal levels of 25OH vitamin D and osteoclastogenesis.

PHASE II.

After the baseline evaluation the patients will be randomly assigned to one treatment group in double blind, the treatment will consist in:

methothrexate (MTX) 15 mg/week im or sc and metilprednisolone (GC) per os 2-4 mg/day (25 patients)

or

MTX 15 mg/week im or sc plus GC 2-4 mg/day plus cholecalciferol 300000 UI at the beginning of the study (25 patients)

After three months of therapy the baseline clinical and experimental evaluations will be repeated. This will allow us to evaluate if the administration of vitamin D improves RA symptoms and has an immunomodulatoy and anti osteoclastogenetic effect.

Statistic analyses

All the statistics will be perform with SPSS 15.0 software.

The unpaired Student's T test will be employ to evaluate the in vitro response to vitamin D by cells from RA patients or from controls.

Patients and controls will be compared for the analysed parameters by ANOVA one way. The immune phenotype, the cytokines production and the osteoclasts formation will be plotted in a multilineare regression models against 25 OH vitamin D, as covariates we will use age and presence or absence of RA will be use as fixed factors. In the patients the model will include the disease activity measurements (VAS, DAS28, quality of life, ESR and RCP).

The paired Student's T test will be employ to evaluate the response to the in vivo administration of vitamin D to RA patients the variables taken into account will be: T cells subsets, cytokines production, osteoclasts precursors number and the disease activity (VAS, DAS28, qualità of life, ESR and RCP).

RESEARCH UNIT DUTIES

the Hospital Unit of Dr Pellerito will:

1. coordinate the project
2. enrol the patients,
3. perform clinical evaluations and clinically follow the patients
4. administer vitamin D and placebo

The University Unit of Prof. Isaia will:

1. enrol the control subjects
2. perform the in vitro experiments on PBMC from RA patients and controls to test the ability of PBMC and T cells to respond to 1,25 OH vitamin D in culture and to metabolize vitamin D by activating or inactivating it
3. perform the ex vivo/in vitro experiments on PBMC from RA patients and controls at baseline (50+50) and after therapy in the patients (50) by FACS, ELISPOT, real time PCR techniques
4. perform the measurements of the inflammatory cytokines produced by PBMC from patients at baseline and after therapy.
5. perform the evaluation of vitamin D intake using Progeo Photo Intake software
6. perform the measurements of vitamin D basal level in serum
7. perform the statistical analysis using SPSS 15.0 software
